# Anti-Infective and Anti-Cancer Properties of the *Annona* Species: Their Ethnomedicinal Uses, Alkaloid Diversity, and Pharmacological Activities

**DOI:** 10.3390/molecules24234419

**Published:** 2019-12-03

**Authors:** Ari Satia Nugraha, Yuvita Dian Damayanti, Phurpa Wangchuk, Paul A. Keller

**Affiliations:** 1 Drug Utilisation and Discovery Research Group, Faculty of Pharmacy, University of Jember, Jember 68121, Indonesia; yuvitadiandamayanti@gmail.com; 2 School of Chemistry & Molecular Bioscience and Molecular Horizons, University of Wollongong, and Illawarra Health & Medical Research Institute, Wollongong, NSW 2533, Australia; 3 Centre for Biodiscovery and Molecular Development of Therapeutics, Australian Institute of Tropical Health and Medicine, James Cook University, Cairns, QLD 4878, Australia; phurpa.wangchuk@jcu.edu.au

**Keywords:** *Annona*, alkaloid, anti-microbial, anti-malaria, anti-protozoa, anti-cancer

## Abstract

*Annona* species have been a valuable source of anti-infective and anticancer agents. However, only limited evaluations of their alkaloids have been carried out. This review collates and evaluates the biological data from extracts and purified isolates for their anti-infective and anti-cancer activities. An isoquinoline backbone is a major structural alkaloid moiety of the *Annona* genus, and more than 83 alkaloids have been isolated from this genus alone. Crude extracts of *Annona* genus are reported with moderate activities against *Plasmodium falciparum* showing larvicidal activities. However, no pure compounds from the *Annona* genus were tested against the parasite. The methanol extract of *Annona muricata* showed apparent antimicrobial activities. The isolated alkaloids from this genus including liriodenine, anonaine, asimilobine showed sensitivity against *Staphylococcus epidermidis.* Other alkaloids such as (+)-Xylopine and isocoreximine indicated significant anti-cancer activity against A549 and K-562 cell lines, respectively. This review revealed that the alkaloids from *Annona* genus are rich in structural diversity and pharmacological activities. Further exploration of this genus and their alkaloids has potential for developing novel anti-infective and anticancer drugs.

## 1. Introduction

*Annona* is one of the 129 genera of the Annonaceae family and contains 119 species with eight species grown for commercial uses [1,2]. Most of the species grow in tropical regions; e.g., the soursop fruit tree (*Annona muricata*) is cultivated commercially and is widespread in the West Indies, North and South Americas, Africa, the Pacific Islands, and Southeast Asia. *Annona* species have been used as medicines by indigenous people for a wide range of disorders including parasitic infections, inflammation, diabetes, and cancer [3]. The phytochemical investigation of this plant genus has revealed the presence of acetogenins, alkaloids, essential oils, flavonoids, terpenoids, and other chemical classes [4,5]. Acetogenins (ACGs) are the major constituents of the *Annona* genre and examples were found to possess a variety of pharmacological properties including as antitumor, immunosuppressive, pesticidal, antiprotozoal, antimicrobial, antimalarial, anthelmintic, and antiviral agents, with some being commercially developed for the treatment of oral herpes and treating infestations of head lice, fleas, and ticks [5,6]. However, the available phytochemistry, including information on the composition and bioactivities of constituents from *Annona* species is limited and scattered [2]. This review evaluates the ethnopharmacological uses, alkaloid constituents, and the anti-infective properties of constituents contained within the genus *Annona*.

## 2. Ethnomedicinal Uses of *Anonna* Genus

The *Annona* species are moderately erect shrubs or small trees that grow to 5–11 m in height depending upon species and the region they inhabit, and are ferruginous to greyish, and tomentose when young, but later becoming glabrous [7]. Ethnobotanically, the plants from this genus play significant roles as food products and medicinal agents. A recent review on *A. muricata* showed that it is widely used in traditional decoctions in as many as 35 different countries for treating numerous diseases [8]; e.g., despite reports that the seed is toxic, traditional Mexican pharmacopeia uses powdered toasted seed as a potent emetic and cathartic. The seed was also used as an insecticidal agent and seed powder was used as a lotion when mixed with grease to treat parasitic skin disorders. A decoction of the fruit skin was used to treat pneumonia [9]. To South-East Asian people, decocted leaves of *Annona reticulate* (“custard apple”) was used internally against worms, and poultice leaves were applied externally to treat abscesses, boils, and ulcers. Unripe fruit was used to treat diarrhea and dysentery, and decocted root was used as febrifuge and to treat toothache [9,10].

In India, *Annona squamosa* (“sugar apple”) leaves are crushed and applied to wounds, ulcers, and is sniffed to relieve hysteria and fainting spells. Decocted leaves are used systemically to treat dysentery (India), and as a tonic, febrifuge, and cold remedy (tropical America). Crushed ripe fruit was applied to surface tumors (India), whereas the unripe fruit was used to treat dysentery in Elsavador [9]. The stem bark and root were used to treat diarrhea and dysentery [9]. The *Annona muricata* (“soursop”) has been used in the indigenous medicine of Togo to treat hypertension and diabetes mellitus [11], with the leaves used as an anti-parasitic, anti-rheumatic, astringent, and emetic in Brazil [12]. Decocted leaves were used as an analgesic, antispasmodic agents in Equador, whereas it is used as a remedy for cough, catarrhal inflammation, diarrhea, dysentery, bladder problems, and inflammation in the West Indies. Mashed leaves were also used as a poultice to relief eczema, rheumatism, and skin eruptions [9]. Traditional medicine in Indonesia has used the leaves as a treatment for boils, spasms, and as an aphrodisiac [13]. The fruit juice was used as a diuretic agent and to treat leprosy and liver ailments [9]. Currently, in Indonesia, the fruit is commonly used traditionally to treat breast cancer. A decoction of the seeds was used as a strong emetic agent, and the flower was used to treat catarrhal inflammation. In Materia Medica of British Guiana, a tincture of the powdered seeds and bay rum serves as a strong emetic. Soursop flowers are believed to alleviate catarrhal inflammation. The roots have been used as a vermifuge and an antidote for poisoning [9]. The roots are commonly used in Guinea as anti-parasitic and pesticidal agents. In Indonesia, currently, the stem and root bark are used as an alternative medication to treat malarial fever.

There are less popular *Annona* species, which were also used in traditional medication. In Guyana, a decoction of the stem bark of *A. ambotay* Aublet was used to treat ulcers and skin eruptions. Mixed with the bark, the leaf was used as febrifuge and sudorific. A tea made of the stem and leaf of *A. glabra* L. was consumed to eliminate flatworm and nematodes in Guyana. A decoction of the bark of *Annona haematantha* Miq. was used as a bath to treat skin ulcers, while its syrup was used to relieve cough. The bark infusion of *Annona sericea* Dunal was used to treat cramps [14]. In Mexico, the leaf of *Annona diversifolia* Safford (“Ilama”) was commonly used as an anticonvulsant, anti-inflammatory, and analgesic agent [15]. An infusion of the leaves of *A. senegalensis* (“wild custard apple”) was used to treat diarrhea and pulmonary complaints. Decocted stem bark was used to treat stomachache, toothache, dysentery, and worm infection. The root was used to treat venereal diseases and intestinal problems, snake bites, and as cancer therapy (Nigeria). Its green fruits was used to treat Guinea worm sores, diarrhea, dysentery [9]. In Brazil, *Annona salzmanii* A. DC has been used to treat dysentery, ulcers, and inflammation [16].

## 3. Phytochemical Studies of Secondary Metabolites of *Annona* Genus

**Figure 1:**
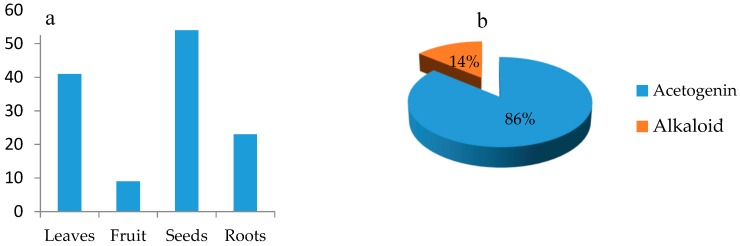
Phytochemical study on *Annona muricata*. (**a**) Number of isolated compounds in different regions of the plants; (**b**) comparison between total isolated acetogenins and alkaloids.

The juicy pulp of the fruit is often a good source of sugar, vitamins, minerals, and phenolic intake. For example, the dried pulp of *Annona muricata* contains 68% sugars for every 100 g containing 1.0 g protein, 0.97 g fat, 1.28 niacin, and 29 mg ascorbic acid. Moreover, it could supply 3 g of phenolic substances for every 100 g of pulp [9,17]. The 20th century reported preliminary examinations of the *Annona* plants of the leaves, fruits, and seeds. Since the 1980s, with the advent of pursuing anti-cancer drug leads from medicinal plants, acetogenin was isolated from the *Annona* genus based on its promising anti-cancer activity. For example, a recent acetogenin, squamocin P, isolated from *A. squamosa,* possessed significant anticancer activity against SMMC 7721/T, MCF-7/ADR, A549/T with IC_50_ values of 0.435, 3.34, 6.32 µM, respectively, with the positive control cisplatin having higher IC_50_ values of 198.85, 178.87, and 219.33 µM against SMMC 7721/T, MCF-7/ADR, and A549/T, respectively. While this encouraged investigations into this species, they were confined to this one polyketide compound, at the expense of other components present. Figure 1a shows the number of compounds isolated from each plant part of *Annona muricata*. In the previous phytochemical studies of *Annona muricata*, around 127 compounds were isolated, in which almost 90% were acetogenins (Figure 1b) [18].

**Figure 2:**
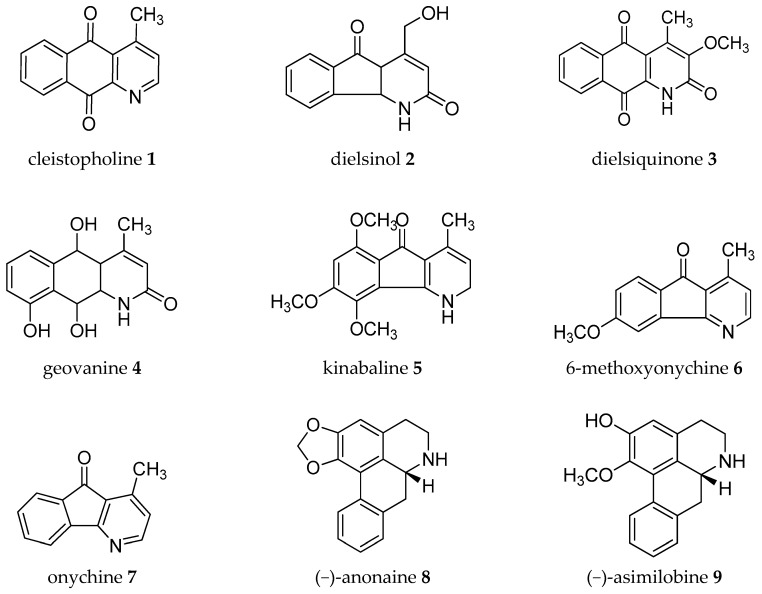

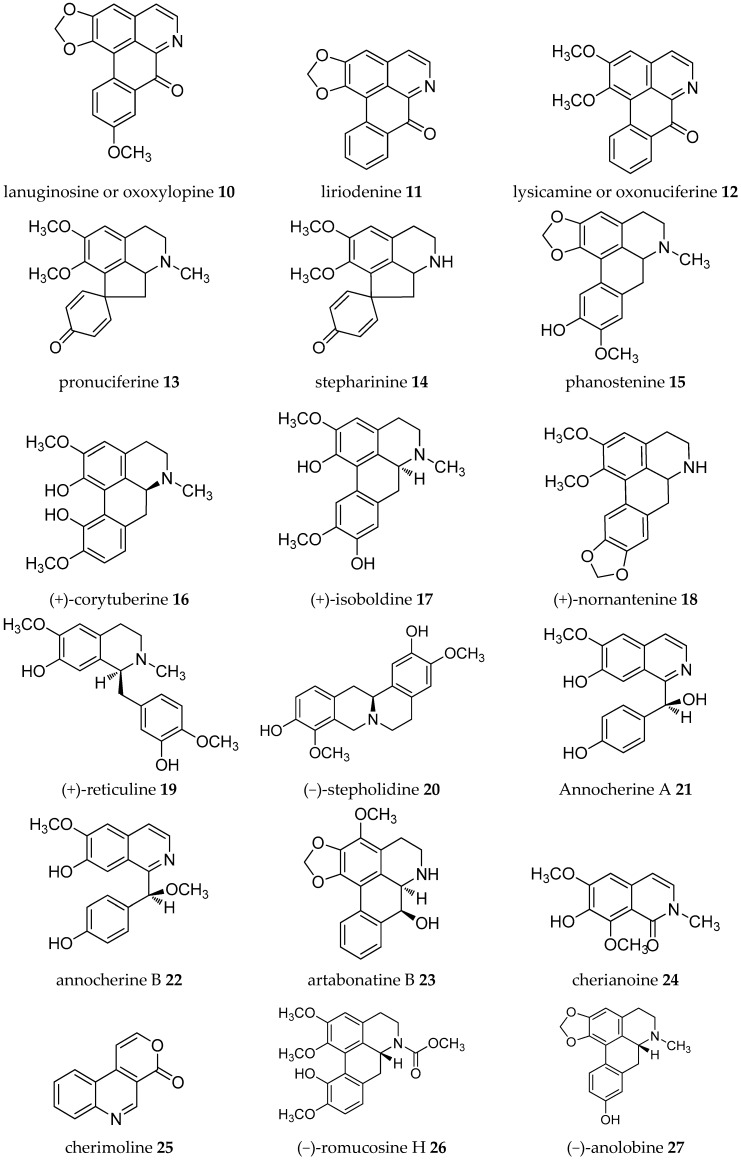

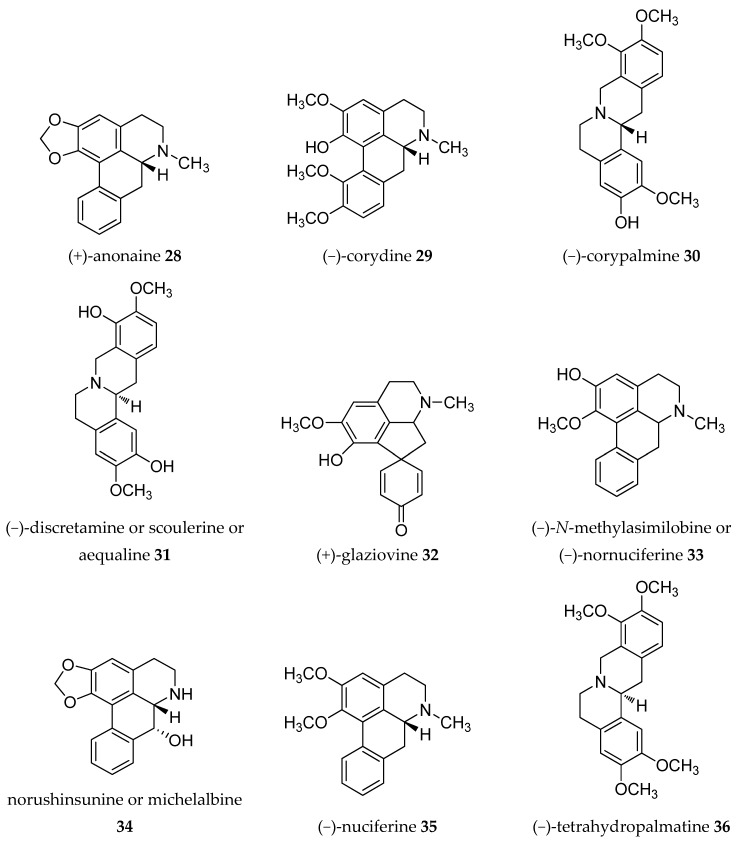

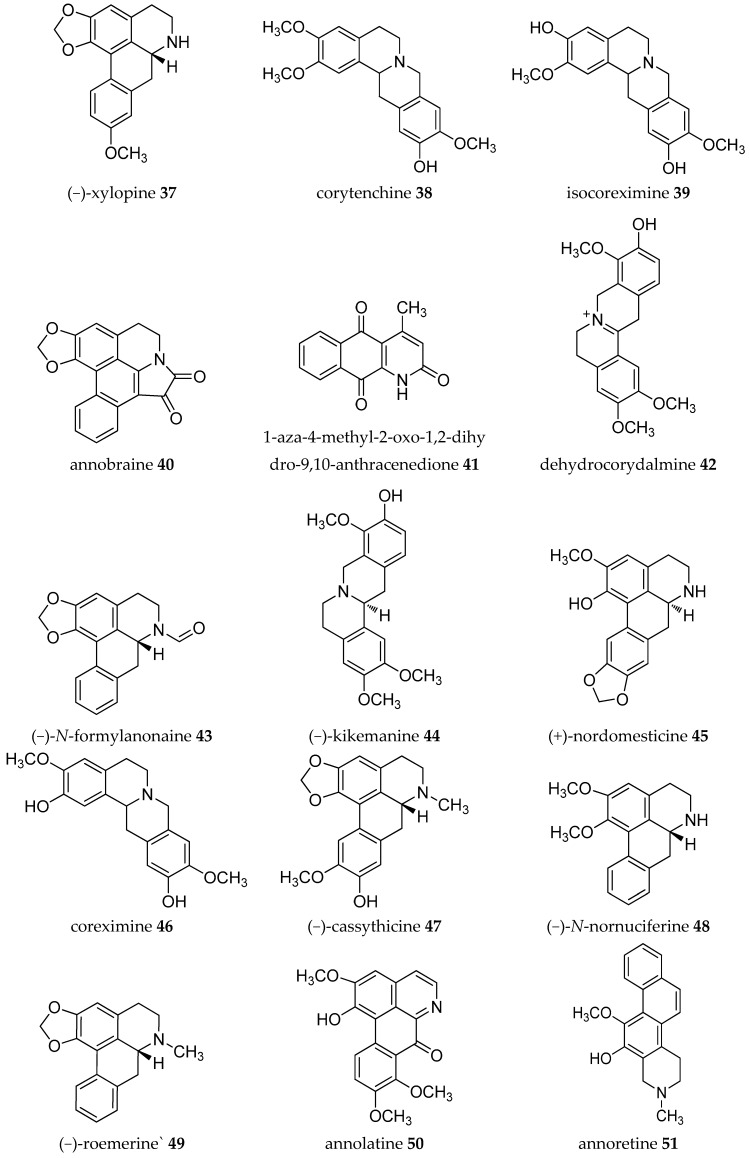

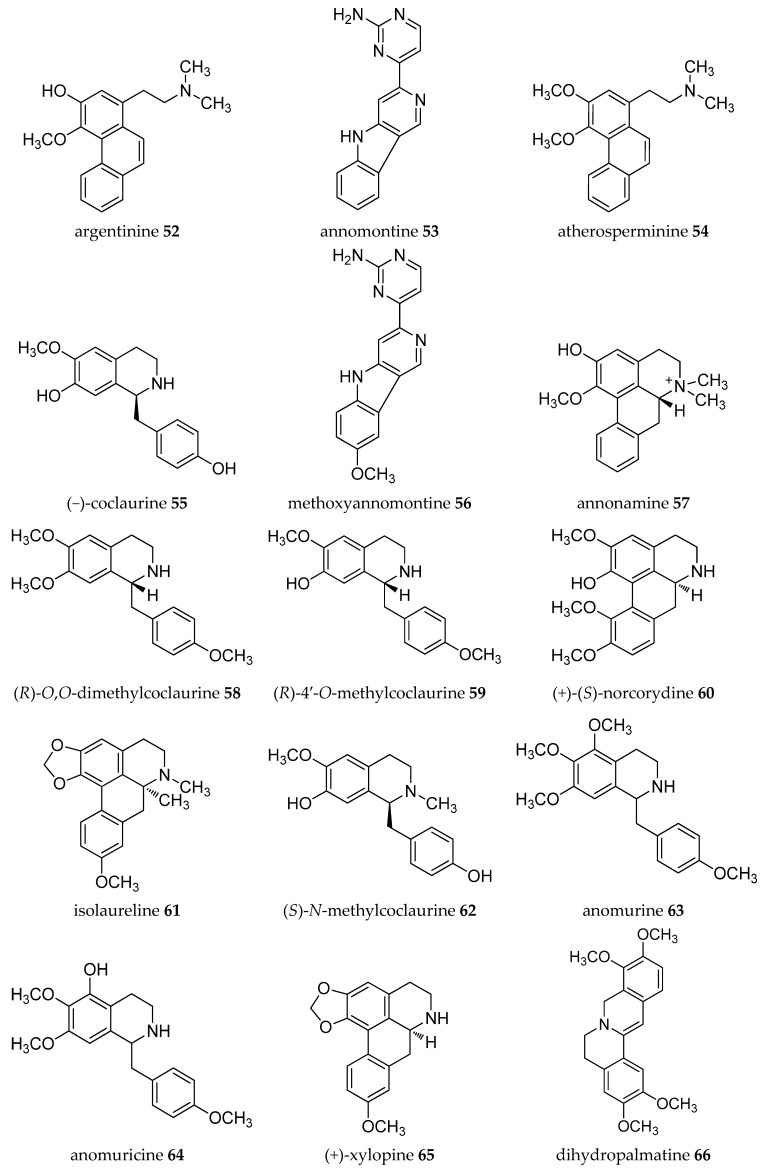

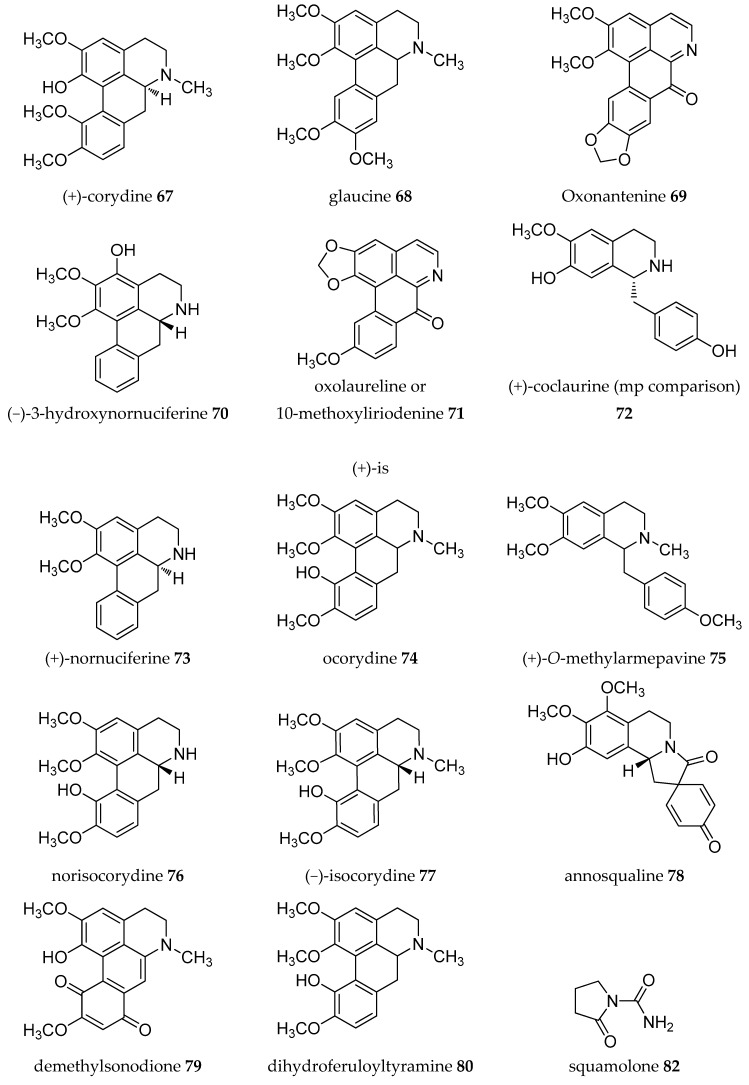

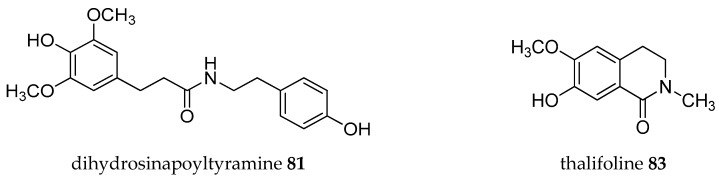
Structures of *Annona* alkaloids (**1**–**83**).

Acetogenins from the *Annona* genus were reviewed together with other genus in the same family Annonaceae [8,19,20,21,22,23], which covered the isolation, molecular properties, and biosynthesis of their pharmacological activities. Here, we collected records on alkaloids which were isolated in the *Annona* plant genus from 1960–2019 (Table 1). The alkaloids present have been of interest since the first, annonaine (**8**, Figure 2), was isolated in 1931 from the stem bark of *Annona muricata* L. collected in the Philippines [24]. Table 1 shows the alkaloids isolated from the specific plants of each species and their structures are presented in Figure 2.

## 4. Anti-Infective Alkaloids from the Genus *Annona*

Plants from the genus *Annona* plants have been used in traditional medication for the treatment of both infectious and non-infectious diseases. This led to the pharmacological and chemical screening of numerous species to confirm these pharmacological claims and to isolate the compounds which might be responsible for these activities. The *Annona* genus has been studied for activity against parasites, cancer, and as anti-oxidant agents.

### 4.1. Antiprotozoal Activities

Ethnopharmacological studies have revealed the *Annona* species *Annona crassiflora*, *A. muricata*, *A. senegalensis*, and *A. squamosa* were prescribed in malarial fever therapy. Further studies revealed leaf extract from *A. crassiflora* was rich in flavonoids and alkaloids, and was able to reduce the *Plamsodium berghei* NK65 infection level in mice by 57–75% with a daily dosage of 12.5 µg/kg/day [62]. Another study of the crude methanol extract of *A. squamosa* indicated moderate activity against *Plasmodium falciparum* 3D7 with an IC_50_ value of 30 µg/mL compared to the chloroquine control, which gave an IC_50_ value of 0.021 µg/mL [63]. Moderate anti-plasmodium activity was also shown using crude extracts of *A. muricata* (Table 2).

In an animal model test, an aqueous leaf extract of *A. muricata* showed a dose dependent antimalarial effect with the highest inhibition of 85.61% observed from a 1000 µg/kg dose. However, the treatment was unable to completely cure the mice, but prolonged the survival time [64]. An essential oil extract of *A. squamosa* demonstrated inhibition against the erythrocitic stages of *P. falciparum,* against epimastigotes forms of *T. cruzi* and against trypomastigotes forms of *T. cruzi* with IC_50_ values of 14.7, 16.2, and 12.7 µg/mL, respectively [65].

Although there was a limited record regarding traditional uses of *Annona* plants to treat other parasitic protozoal infections, e.g., leishmaniasis and trypanosomiasis, several crude extracts of *Annona* plant (*A. muricata*) were also tested against *L. amazonensis*, *L. braziliensis*, *L. Donovani,* and *T. cruzi* (Table 2). The crude ethyl acetate extract from the leaves of *A. muricata* indicated potent activity against *L. amazonensis*, *L. braziliensis*, *L. Donovani*, and *T. cruzi* with IC_50_ values of 10–25 µg/mL. A different strategy to control malarial infection involves controlling its vector. Past larvicidal studies have indicated that the crude methanol extract from the bark of *A. squamosa* resulted in 100% mortality of *Anopheles subpictus* (which carry human malaria parasites) at 500 µg/mL [66]. The extract from the stem and root bark were even more toxic toward malarial larvae (*Anopheles gambiae*s.s. Giles) with 50% mortality at 24 and 21 µg/mL, respectively [67].

The same protocol was applied to other disease vectors, including Aedes (dengue virus vector) and Culex (encephalitis virus). For example, the seeds of *Annonas* pecies were generally reported to be toxic with LC_50_ values <1 µg/mL against both Aedes and Culex larvae (Table 3). These results demonstrated that the *Annona* plants can be used for controlling the vector especially in rural areas where modern, and likely more expensive, vector controls were limited.

Despite numerous alkaloids being isolated from *Annona* species, reports detailing pharmacological studies on single compounds remains limited. There are reports on the same alkaloids being isolated from different plant genus. For example, (+)-reticuline **19** was isolated from *Croton linearis* and was previously shown to possess a weak antriprotozoal activity against *Lesihmania infatum* with IC_50_ values of 148.0 ± 1.2 µM [70]. Asimilobine **9** and isoboldine **17** isolated from the bark of *Beilschmiedia alloiophylla* (Costa Rica) possessed anti-leishmanial activity with IC_50_ values of 29.8 ± 1.5 µM and 50.0 ± 4.0 µM, respectively [71]. A previous study on the leaves and fruits of *Annona mucosa* (Brazil) produced liriodenine **11**, which was highly active against *Leishmania amazonensis* with an IC_50_ value of 1.43 ± 0.58 μg/mL and was moderately active against *Leishmania braziliensis* with an IC_50_ value of 55.92 ± 3.55 μg/mL [72].

### 4.2. Antimicrobial Activities

Traditionally, *Annona* plants have been prepared for use against infection related diseases, such as ulcer, dysentery, and boils, and therefore became a driving force for conducting anti-microbial studies against common bacteria; preliminary results on the crude extracts are shown in Table 4. In general, the crude extract possessed moderate to inactive anti-microbial values ranging from 6.25–4096 µg/mL. Most of the studies were based on the anti-microbial activity of crude extracts with no separate non-polar to polar fractions tested or individual constituents isolated. Therefore, further investigations are required to substantiate the traditional claims for these *Annona* plants by the isolation and identification of individual constituents. As a result, discussion here is confined to the anti-microbial activities from isolated alkaloid constituents (Table 5). *A. muricata, A. squamosa, A. cherimola, and A. ambotay* showed reasonable antimicrobial activities, whereas *A. reticulata* did not present antimicrobial activity, with reported MIC values of more than 1000 µg/mL against *Bacillus cereus*, *Staphylococcus aureus* [77]. Antimicrobial testing of the methanol extract of *A. squamosa* fruit against multidrug resistant MRSA reported MIC values of 5000 µg/mL, but no information was given against ESBLEC (extended-spectrum beta-lactamase producing *E. coli*), CRPA (carbapenem-resistant *P. aeruginosa*) and MDRAB (multidrug-resistant *A. baumannii*) [78]. The benzoquinoline alkaloid, anonaine **8**, indicated comparable anti-microbial activities with positive control, with the exception against *Staphylococcus aureus*. Another study reported annoquinone A, isolated from *A. Montana*, possessed anti-microbial activity against *Bacillus subtilis* and *Micrococcus luteus* with IC_50_ value of 10, 10 µg/mL, respectively [79].

Previous studies using alkaloid samples from sources other than *Annona* revealed, (−)-asimilobine **9** isolated from the bark of *Beilschmiedia alloiophylla* (Costa Rica) and *B. kunstleri* (Malaysia) indicated anti-fungal activity with an IC_50_ value of 16.0 µg/mL [71]. (−)-Stepholidine **20** isolated from rattan stem of *Fibraurea recisa* had antifungal activity against drug resistant *Candida albicans* SM372, *Candida krusei* KM066, *Candida parapsilosis* SM304160, *Cryptococcus neofarms* SM9406204 with similar MIC value of 320 µg/mL [91]. Alkaloid (−)-roemerine **49** from the same stem indicated significant inhibition of *C. albican* transition from yeast to hyphae in a dose dependent manner [92]. Glaucine **68** isolated from the aerial component of *Glaucium oxylobum* showed moderate skin anti-fungal activities against *Microsporum canis*, *Microsporum gypseum,* and *Trichophyton mentagrophytes* [93]. Antifungal activities of the non-*Annona* isolated alkaloids were evaluated against non-pathogenic fungi including liriodenine **11** from the wood of *Michelia formosa* which indicated a low activity against several wood decaying fungi both white and brown rot-fungi, *Lenzites betulina*, *Trametes versicolor*, *Laetiporus sulphureus*, *Gloeophyllum trabeum,* and *Fomitopsis pinicola* [94]. Similar alkaloids were also previously evaluated against pathogenic bacteria, including liriodenine from the roots of *Zanthoxylum nitidum* which showed a good antimicrobial activity against MRSA with MIC value of 93.8 µg/mL [95]. Liriodenine **11** from the stem of *Mitrephira glabra* Scheff was active against non-pathogenic bacteria, *Micrococcus luteus*, *Mycobacterium sinegmatis*, *Saccharomyces cerevisae*, and *Aspergilus niger* with an MIC value of 6.3, 12, 12, and 25 µg/mL, respectively [96].

## 5. Anticancer Alkaloids Present in the Genus *Annona*

In addition to the above antiprotozoal and antimicrobial activities, both the crude extracts from *annona* plants and the individual alkaloids have shown potent anticancer/antitumour activities.Many crude extracts of *Annona* species showed significant anti-cancer activities, but most of the bioactive constituents present in those crude extracts were acetogenins, fatty acids, and peptides [7]. However, wherever studied, it was known that some aporphine alkaloids, especially (−)-roemerine **49**, which was isolated from the leaves of the wild custard apple, improved the response produced by vinblastine against multidrug-resistant KB-V1 or KB-3 cells (ED_50_ > 20 µg/mL). This alkaloid appears to function by interacting with P-glycoprotein in the multidrug-resistant KB-V1 cell membrane vesicles [59]. The leaves of *Annona muricata* also showed potency to reduce gastric lesion, to expel parasitic worms and, moreover, the crude extract from the bark possessed anti-viral activity against herpes simplex virus type 1. The extracts and compounds also showed anticancer activities against breast cancer. Alkaloids, (−)-coclaurine **55**, (+)-reticuline **19**, argentinine **52**, atherosperminine **54**, and (+)-xylopine **65** were isolated from the root of Indonesian *Annona muricata* in which (−)-coclaurine **55**, (+)-reticuline **19** were non-toxic against a human suspension cancer cell line (HL-60 leukemia cells) and two fibroblastic cell lines (A549 lung cancer cells and HepG2 liver cancer cells). (+)-Xylopine **65** exhibited the lowest IC_50_ value ranging from approximately 20–80 µM [18].The alkaloid isocoreximine **39** isolated from *Annona cherimola*, at concentration of 50 µg/mL indicated cytotoxicity against K-562, U-251, PC-3, HCT-15, and MCF-7 with % inhibition of cell viability 94.15%, 65.23%, 78.71%, 63.05%, and 85.76%, respectively. Isocoreximine **39** showed in vitro cytotoxic activity against K-562, U-251, PC-3, HCT-15, and MCF-7 with % of inhibition of cell viability 94.15%, 65.23%, 78.71%, 63.05%, and 85.76%, respectively [34].

Although most of the alkaloids isolated from *Annona* species were reported with no anticancer activity data, there were cytotoxicity activity data on similar molecules obtained from non-*Annona* genus (Table 6). Interestingly, annomontine **54**, a carbolated pyrimidine alkaloid was previously reported from the marine sponge *Acanthostrongylophora ingens* collected from Indonesian water. The alkaloid possessed pronounced anticancer activity against mouse lymphoma L5178Y compared to a standard control kahallide F [97]. The oxoaporphine alkaloid, liriodenine **11**, was found in at least in twenty different species, ranging across flowering plants but mostly in annonaceae family. The alkaloid isolated from Brazilian *Guatteria blepharophylla* stem bark possessed anticancer activity against MCF-7 cell line with a more potent result compared to a standard drug doxorubicin with TGI value of 36.67 compared to 46.04 µM [98].

## 6. Conclusions

This review presents the ethnomedicinal, alkaloidal and biological, properties of *Annona* species with respect to reported anti-infective and anti-cancer activities. The *Anonna* species: *A. muricata* (soursop), *A. squamosa* (custard apple), *A. senegalensis* (wild custard apple), and *A. cherimola* (cherimola) are renowned traditionally for their anti-tumor properties. Among these, *A. muricata* is widely studied and has shown broad range of biological activities including anti-protozoal, anti-cancer, anti-tumour, antimicrobial, and antiparasitic properties. This species has also produced several patents and commercial products. Investigations into extracts from the leaves, bark, fruit, and seeds of this plant genus have found terpenoids, steroids, flavonoids, cardiac glycosides, tannins, phenols, sugars, fatty acids, acetogenins, and alkaloids. As many as 200 phytochemicals belonging to these chemotypes have been identified and isolated from this *Annona muricata* species alone, with the most important being acetogenins, phenols and anonaine alkaloids. Anonaine and its structurally related alkaloids were the most abundant and commonly available alkaloids in *Annonaceae* family. The oxoaporphine alkaloid, liriodenine, was found in at least in twenty different species, ranging across flowering plants but mostly in annonaceae family. The alkaloids from *Annona* species have rarely been explored for their medicinal applications. However, wherever studied, *Annona* alkaloids have been reported to possess anti-inflammatory, anti-cancer, antitumor, anti-HIV, antiprotozoal, antiparasitic, antidiabetic, analgesics, gastroprotective, antihypertensive, hepatoprotective, nephroprotective, and neuroprotective properties. Amongst these broad-ranging properties, anti-cancer and anti-tumour activities of both the crude extracts and alkaloids is commendable. Most interesting and noteworthy of this *Annona* genera is that the pharmacological properties accentuate the ethnomedicinal utilization of this plant, as well as its usefulness in the agrifood sector. Liriodenine, annonaine, glaucine and cleistopholine showed potent anti-cancer, anti-tumour, and cytotoxicity activities against many human cancer cell lines, and it is worthwhile to pursue detailed clinical investigations of these alkaloids. To the best of our knowledge, there is no clinical study that was successfully completed on the extracts rich in acetogenins or alkaloids. In this respect, it is also necessary to conduct scientific studies to establish optimal and safe doses of consumption of both the plant extracts and their phytochemicals especially alkaloids. This is because the use of the *Annona* plants is popular not only in Indonesia, but wide across the tropical countries.

## Figures and Tables

**Table 1:** Alkaloid Constituents of *Annona*.

Plant Parts	Location	Isolated Alkaloids
*Annona ambotay*		
Wood	Brazil	benzene-EtOH: cleistopholine **1**, dielsinol **2**, dielsiquinone **3**, geovanine **4**, kinabaline **5**, 6-methoxyonychine **6**, onychine **7** [25]
*Annona cherimola*		
Leaves	Brazil	(−)-anonaine **8**, (−)-asimilobine **9**, lanuginosine **10**, liriodenine **11**, lysicamine **12**, pronuciferine **13**, (+)-stepharine **14** [26]
Leaves	India	Phanostenine **15** [27]
Leaves	Spain	(−)-anonaine **8**, (+)-corytuberine **16**, (+)-isoboldine **17**, lanuginosine 10, liriodenine **11**, (+)-nornantenine **18**, (+)-reticuline 19, (−)-stepholidine **20** [28]
Seeds	Spain	(−)-anonaine **8**, cleistopholine **1**, lanuginosine **10**, liriodenine or xoushinsunine **11** [29,30]
Stem	Taiwan	(+)-annocherine A **21**, (+)-annocherine B **22**, (−)-artabonatine B **23**, cherianoine **24**, cherimoline **25**, (−)-romucosine H **26** [31,32]
Stem	Spain	(−)-anolobine **27**, (+)-anonaine **28**, (−)-asimilobine **9**, (−)-corydine **29**, (−)-corypalmine **30**, (−)-discretamine **31**, (+)-glaziovine **32**, (+)-isoboldine **17**, lanuginosine **10**, liriodenine **11**, lysicamine **12**, (−)-*N*-methylasimilobine **33**, (−)-norushinsunine **34**, (−)-nuciferine **35**, (−)-stepholidine **20**, (−)-tetrahydropalmatine **36**, (−)-xylopine **37**, (+)-reticuline **19** [33]
Root	Mexico	(−)-corytenchine **38**, (−)-isocoreximine **39** [34]
*Annona diversifolia*		
Roots	Mexico	Liriodenine **11** [35]
*Annona glabra*		
Fruit-stem	Taiwan	(−)-anonaine **8**, annobraine **40**, (−)-asimilobine **9**, 1-aza-4-methyl-2-oxo-1,2-dihydro-9,10-anthracenedione **41**, dehydrocorydalmine **42**, (−)-*N*-formylanonaine **43**, (−)-kikemanine **44**, liriodenine **11**, lysicamine **12**, (−)-nornuciferine or (−)-*N*-methylasimilobine **33**, (+)-nordomesticine **45**, (+)-stepharine **14** [36]
Leaves	Mexico	(−)-anonaine **8**, asimilobine **9**, coreximine **46**, (+)-reticuline **19** [37]
Leaves	Taiwan	(−)-*N*-methyl-actinodaphnine **47**, (+)-reticuline **19** [38]
Root	Mexico	(−)-anonaine **8**, (−)-asimilobine **9**, (−)-coreximine **46**, (−)-nornuciferine or (−)-*N*-methylasimilobine **33**, (+)-reticuline **19** [37]
Stem	Mexico	(−)-anonaine **8**, (−)-asimilobine **9**, (−)-nornuciferine or (−)-*N*-methylasimilobine **33**, (+)-reticuline **19** [37]
Stem	Taiwan	(−)-anolobine **27**, (−)-anonaine **8**, (−)-asimilobine **9**, (+)-isoboldine **17**, liriodenine (or oxoushinsunine) **11**, (−)-*N*-nornuciferine **48**, (−)-norushinsunine (or michelalbine) **34**, (+)-reticuline **19**, (−)-roemerine **49** [39,40]
*Annona montana* Macf (wild soursop)		
Leaves	Taiwan	annolatine **50**, annoretine **51**, argentinine **52**, liriodenine **11** [41]
Stem-Root bark	Guinea	Annomontine **53**, (−)-anonaine **8**, atherosperminine **54**, (−)-asimilobine **9**, (−)-coclaurine **55**, (−)-coreximine **46**, methoxyannomontine **56**, oxoushinsunine or liriodenine **11**, (+)-reticuline **19**, (−)-xylopine **37** [42]
Stem bark	Japan	Annomontine **53** [43]
*Annona muricata* L. (soursop)		
Leaves	Tanzania	(−)-anonaine **8**, (−)-roemerine **49** [44]
	Japan	(−)-anonaine **8**, (−)-annonamine **57**, (*+*)-*O*,*O*-dimethylcoclaurine **58**, (*+*)-4′-*O*-methylcoclaurine **59**, (*+*)-norcorydine **60** [45]
Leaves	Guinea	(−)-anonaine **8**, (−)-coclaurine **55**, isolaureline **61**, isoboldine **17**, liriodenine **11**, (+)-*N*-methylcoclaurine **62**, norisolaurelin or (−)-xylopine **37**, (−)-roemerine **49** [46,47]
Stem (bark)	Guinea	Anomurine **63**, anomuricine **64**, atherosperminine **54**, (−)-coclaurine **55**, (−)-coreximine **46**, (+)-reticuline **19**, (+)-stepharine **14** [48]
Roots	Indonesia	(−)-coclaurine **55**, (+)-reticuline **19**, argentinine **52**, atherosperminine **54**, (+)-xylopine **65** [18]
*Annona paludosa* Aubl.		
Root bark	Guiena	(−)-anonaine **8**, (−)-asimilobine **9**, (−)-coreximine **46**, dihydropalmatine **66**, (+)-reticuline **19**, (−)-scoulerine or (−)-discretamine **31**, (−)-roemerine **49**, (±)-tetrahydropalmatine **36** [49]
*Annona reticulata*		
Leaves	Taiwan	(−)-asimilobine **9**, (+)-corydine **67**, (+)-glaucine **68**, liriodenine **11**, (+)-norcorydine **60**, oxonantenine **69**, oxoxylopine or lanuginosine **10**, (−)-xylopine **37** [50]
Roots	Taiwan	(−)-aequaline or (−)-discretamine **31**, (+/-)-annomontine **53**, (−)-anonaine **8**, (−)-asimilobine **9**, (−)-3-hydroxynornuciferine **70**, liriodenine **11**, methoxyannomontine **56**, (−)-michelalbine or (−)-norushinsunine **34**, oxoushinsunine or liriodenine **11**, (+)-reticuline **19** [51,52]
*Annona salzmanii* A. DC		
Bark	Brazil	(−)-anonaine **8**, (−)-asimilobine **9**, cleistopholine **1**, liriodenine **11**, oxolaureline or 10-methoxyliriodenine **71**, (+)- reticuline **19**, (−)-xylopine **37** [16]
*Annona sericea*		
Leaves		(−)-3-hydroxynornuciferine **70**, (+)-isoboldine **17**, (+)-*N*-methylcoclaurine **62**, (+)-nornantenine **18**, (−)-nornuciferine or (−)-*N*-methylasimilobine **33**, oxonuciferine or lysicamine **12**, (+)-reticuline **19** [53]
*Annona squamosa*		
Leaves	Brazil	(−)-anonaine **8**, asimilobine **9**, liriodenine **11**, (−)-nornuciferine or (−)-*N*-methylasimilobine **33**, (+)-reticuline **19** [54]
Leaves- stem bark	Guinea	(−)-anonaine **8**, (+)-coclaurine **72**, (+)-isoboldine **17**, liriodenine **11**, (+)-nornuciferine **73**, (−)-roemerine **49** [55,56]
Leaves	India	(−)-anonaine **8**, (+)-corydine **67**, (+)-glaucine **68**, (+)-isocorydine **74**, lanuginosine **10**, (+)-*O*-methylarmepavine **75**, (+)-norcorydine **60**, norisocorydine **76**, (−)-roemerine **49**, (−)-xylopine **7** [57,58]
Leaves	Tanzania	(−)-anonaine **8**,(−)-roemerine **49** [44]
Leaves	Zimbabwe	(−)-isocorydine **77**, (−)-roemerine **49** [59]
Seeds	Brazil	(−)-anonaine **8**, asimilobine **9**, corypalmine **30**, (−)-nornuciferine or (−)-*N*-methylasimilobine **33**, (+)-reticuline **19** [54]
Stem	Taiwan	Annobraine **40**, annosqualine **78**, demethylsonodione **79**, dihydroferuloyltyramine **80**, dihydrosinapoyltyramine **81**, liriodenine **11**, squamolone **82**, thalifoline **83** [60]
Roots	Taiwan	(−)-anolobine **27**, (−)-anonaine **8**, (−)-norushinsunine (or michelalbine) **34**, oxoushinsuine (liriodenine) **11**, (+)-reticuline **19** [61]

**Table 2:** Anti-protozoal activity of several extract of *Annona muricata* and *Annona reticulata* [68,69].

Species	Part of Plant (Extract)	Anti-Protozoal Activity (IC_50_, µg/mL)					
		*Leishmania species*			*Trypanosoma cruzi*	*P. falciparum*	
		PH8	M2903	PP75		F32	W2
*A. muricata*	LF (Hexane)	100.0	>100.0	>100.0	100.0	7.2 ^a^	38.6 ^a^
	LF (EtOAc)	25.0	25.0	25.0	25.0	8.5 ^a^	10.4 ^a^
	LF (MeOH)	>100.0	>100	>100.0	100.0	9.2 ^a^	36.8 ^a^
	SD (Hexane)	98.6	76.3	83.1	74.9	11.4 ^a^	38.2 ^a^
	SD (EtOAc)	63.2	63.2	63.2	63.2	40.2 ^a^	34.7 ^a^
	SD (MeOH)	98.6	98.6	98.6	98.6	32.5 ^a^	26.3 ^a^
	PC (EtOH)						1.01
	PC (H_2_O)						>10
	PC (CH_2_Cl_2_)						0.94
	RT (EtOH)						0.79
	RT (H_2_O)						>10
	RT (CH_2_Cl_2_)						0.19
	ST(EtOH)						1.45
	ST (H_2_O)						>10
	ST (CH_2_Cl_2_)						3.32
*A. reticulata*	LF(EtOH)						>10
	LF (H_2_O)						>10
	LF (CH_2_Cl_2_)						>10
	TW (EtOH)						>10
	TW (H_2_O)						>10
	TW (CH_2_Cl_2_)						0.88
	ST(EtOH)						0.29
	ST (H_2_O)						>10
	ST (CH_2_Cl_2_)						0.82
	RT (EtOH)						1.90
	RT (H_2_O)						>10
	RT (CH_2_Cl_2_)						0.38
	FR (EtOH)						0.67
	RF (H_2_O)						>10
	RF (CH_2_Cl_2_)						0.42
Standard drug	Pentamidine	10.0	10.0	10.0			
	Amphotericin B	0.2	0.2	0.2			
	Bensoidazole				2.0		
	Chloroquine					0.01	0.9
	Artemisisn						0.005

LF: leaf; SD: seed, PC: pericarp; RT: root; ST: stem bark; TW: twig; *Leishmania amazonensis* (PH8); *Leishmania braziliensis* (M2903); *Leishmania donovani* PP75; ^a^ Values represent percentage of inhibition at 10.0 µg/mL.

**Table 3:** Larvicidal of several extract of *Annona* genus [44,66,73,74,75,76].

Plant Name	Plant Extract	LC_50_ (µg/mL)			
		*Aedes aegypti*	*Aedes albopictus*	*Culex quinquefasciatus*	*Culex tritaeniorhynchus*
*A. crassiflora*	SB (hexane)	192.57			
	RW (hexane)	154.02			
	RB (hexane)	264.15			
	RB (EtOH)	0.71			
	RW (EtOH)	8.94			
	ST (EtOH)	16.1			
*A. glabra*	SD (EtOH)	0.06			
*A. muricata*	RT (EtOH)	42.3			
	SD (hexane)	122.77			
	SD (CHCl_3_)	0.90			
	SD (MeOH)	85.91			
	LF (MeOH)			56.47	
*A. senegalensis*	LF (MeOH)			23.42	
*A. squamosal*	RT (EtOH)	31.9			
	LF (EtOH)	169	20.70		
	SD (EtOH)	5.12	6.96		
	LF (MeOH)		20.26	17.70	
	SB (MeOH)				104.94

SB: stem bark; RB: root bark; RW: root wood; SD: seed; RT: root; LF: leaf.

**Table 4:** Anti-microbial activities of crude extracts or fractions of *Annona* genus.

Plant Name/Standards	Plant Extract	MIC (µg/mL)																				
		ST	PA	KP	BC	EC	SA	PS	XC	AT	PM	PC	EH	TV	NB ^a^	MD ^a^	BC ^b^	AN ^c^	AI ^c^	SM ^c^	PI ^c^	PG ^c^
*A. ambotay* [80]	LF (EtOH)						9 b										10 b					
*A. cherimola* [81]	SD (MeOH)												>100	15	5	8						
*A. cherimola* [80]	LF (EtOH)						11b										14b					
*A. muricata* [82,83,84]	LF (H_2_O)	4096	1024	512		>1024	>1024															
*A. muricata* [81]	SD (MeOH)												>100	30	26	25						
*A. muricata* [85]	SB (EtOH)		6.25	6.25	12.5																	
*A. muricata* [80]	STm (EtOH)																					
*A. muricata* [18]	RT (MeOH)		>32			>32	>32															
*A. squamosa* [86]	SD (EtOH)							>771	>771	>771	>771	>771										
*A. squamosa* [86]	SD (Acetone)							>475	>475	>475	>475	>475										
*A. squamosa* [87]	SD (MeOH)		50 *			50 *	50 *															
*A. squamosa* [78]	FR (MeOH)				1250 *	1250 *	1250 *															
*A. Senegalensis* [88]	BK (MeOH)																	4.5	5.0	3.0	2.5	6.5
Streptomycin								10	10	20	10											
Chloramfenicol							RST										30					
Metronidazole													1.25	2.5								
Ivermictine															0.8	1.3						
Neomycin						312.5	312.5															
Gentamycin			0.06	0.06	0.01		0.12															

RST: Resistance; ^a^ LD_50_ (µg/mL); ^b^ Inhibition zone (0.1 mg/disc, mm); ^c^ Inhibition zone (2 mg/disc, mm); LF:Leaf; SD: Seed; BK: Bark; STm: Stem; ST: *Salmonella typhi*; PA: *Pseudomas aeruginosa*; KP: *Klebsiella pneumonia*; EC: *Escherichia coli* 27; SA: *Staphylococcus aureus* 358; PS: *Pseudomonas syringa* e673; AT: *Agrobacterium tumefaciens* 431; XC: *Xanthomonas campestris* 2286; PC: *Pectobacterium caratovorum* 1428; PM: *Pseudomonas marginalis* 2758; AP: *Aspergillus parasiticus* 411; EH: *Entamoeba histolytica*; TV: *Taenia vaginalis*; NB: *Nippostrongylus brasiliensis*; *Mollemades setae*; BC: *Bacilus subtilis;* AN: *Actinomyces naeslundii*; AI: *Actinomyces israelii*; SM: *Streptococcus mutans*; PI: *Privotella intermedia*; PG: *Porphyromonus gingivalis*. * MIC was recorded in µg /mL.

**Table 5:** Anti-microbial activities of alkaloids isolated from *Annona* genus.

Compound		MIC (µg/mL)											
	KZ	SA	Sap	SE	Sep	EF	EC	PA	CA	CP	CD	CDb	Fm
*A. salzmannii* [89]													-
Liriodenine 11	-	>500	>500	50	50	-	-	-	-	-	50	100	-
Anonaine 8	50	>500	50	25	50	-	-	-	-	-	50	50	-
Asimilobine 9	50	>500	50	50	50	100	-	-	>500	>500	>500	50	-
Reticuline 19	250	>500	>500	100	100	250	-	-	100	100	>500	>500	-
*Annona squamosal* [90]													
(−)-(*R*)-anonaine 8 [90]	-	-	-	-	-	-	-	-	-	-	-	-	30–39 *
Cleistopholine 1	-	-	-	250	250	250	-	-	-	-	-	250	-
Chloramphenicol	50	25	25	50	50	50	50	850	12.5	12.5	12.5	12.5	-

-: no data available; KZ: Kocuriarhizophila (ATCC 9341); SA: Staphylococcus aureus (ATCC14458); SAp:S. aureuspenicilinase-(8-); SE: Staphylococcus epidermidis (ATCC 12228); Sep; S. epidermidis (6ep); EF: Enterococcus faecalis (Ef); EC: Escherichia coli (ATCC 10538); PA: Pseudomonas aeruginosa (ATCC 27853)c; CA: Candida albicans (ATCC 10231); CA: Candida albicans(ATCC 10231); CP: Candida parapsilosis (ATCC 22019); CD: Candida dubliniensis (ATCC 777); CDb Candida dubliniensis (ATCC 778157); FM: Fusarium moniliforme. * inhibition diameter in mm.

**Table 6:** Anticancer/cytotoxicityactivities ofalkaloids that were obtained from non-*Annona* genera.

Alkaloid	Plants	Part of Plant	Country	Anticancer Activity	Ref(s)
(−)-Anonaine **8**	*Nelumbo nucifera* Gaertn (Nelumbonaceae)	Leaves	Taiwan	Anti-proliferative effects with IC_50_ > 500 µM against AGS and 150.1 ± 0.3 µM against DU-145	[99]
	*Michelia alba* D.C. (Magnoliaceae)	Leaves	Taiwan	Inhibited viability of HeLa cancer cells (23 ± 1%) more effectively than non-cancer cells (Vero and MDCK cells, 75 ± 3% and 95 ± 4%, respectively) at concentration of 100 µM.	[100]
Annomontine **53**	*Acanthostrongylophora ingens* (Petrosiidae)	Sponges	Indonesia	Pronounced cytotoxicity against L5178Y cell line with ED_50_ 7.8 µg/mL compared to the positive control kahalalide F (ED_50_ 6.3 μg/mL)	[97]
	*Acanthostrongylophora ingens* (Petrosiidae)	Sponges	Indonesia	Pronounced cytotoxicity against L5178Y cell line with EC_50_ 0.49 µg/mL	[101]
Artabonatine B **23**	*Artabotrys hexapetalus* (L.f.) Bhandari (Annonaceae)	Roots, stems, and leaves	Taiwan	Active against both Hep G2 and 2,2,15 cell lines with IC_50_ 9.1 and 11.0 µg/mL, respectively	[102]
(−)-Asimilobine **9**	*Nelumbo nucifera* Gaertn (Nelumbonaceae)	Leaves	Taiwan	Anti-proliferative effects against AGS and DU-145 cell lines with IC_50_ > 500 µM	[99]
Cleistopholine **1**	*Cananga odorata* (Lam.) Hook.f. & Thomson (Annonaceae)	Fruits	Taiwan	Displayed potent cytotoxicity against Hep G2 (human hepatoma cell) and Hep 2,2,15 (Hep G2 cell line transfected with hepatitis B virus) cell lines with IC_50_ value of 0.22 µg/mL and 0.54 µg/mL, respectively	[103]
	*Disepalum pulchrum* (King) J.Sinclair (*Enicosanthellum pulchrum*, Annonaceae)	Roots	Malaysia	Active against CAOV-3 and SKOV-3 with IC_50_ value of 61.4 μM and 67.3 μM, respectively. This was comparable with that of the positive control cisplatin (62.8 μM and 67.1 μM) at 24 h of treatment. Cleistopholine (1) at >200 μM showed less cytotoxic effect against normal ovarian cells (SV40).	[104]
	*Saprosma hainanense* Merr. (Rubiaceae)	Stems	China	Inactive against against BEL-7402, SGC-7901, and K-562 cell lines	[105]
(−)-Corydine **29**	*Dicranostigma leptopodum* (Maxim.) Fedde (Papaveraceae)	Whole plant	China	Showed its cytotoxicity against H1299, MCF-7, and SMCC-7721 with IC_50_ > 100 µM	[106]
	*Stephania dinklagei* (Engl.) Diels (Menispermaceae)	Aerial parts	Ghana	Exhibited cytotoxic activity against KB cell line with IC_50_ 733 μM	[107]
(−)-Corydine **29**	*Stephania dinklagei* (Engl.) Diels (Menispermaceae)	Stem	Ghana	(−)-Corydine 29 showed DNA-damaging activity in the yeast bioassay (IC50 values YCp50 gal, pRAD52 GAL, Prad52 GLU were27.5, >73.9, and 22.5 μg/mL, respectively	[108]
	*Stephania kwangsiensis* H.S. Lo. (Menispermaceae)	Root	India	Three different concentrations (20, 10, 5 µg/mL) could all significantly increase the apoptosis rate (8.77%, 9.12%, and 12.38%, respectively) of NCI-H446 cells after 48 h of treatment compared to the control group (1.02%). (−)-Corydine 29 can inhibit the proliferation of lung cancer NCI-H446 cells and induce their apoptosis	[109]
Corytuberine **16**	*Dicranostigma leptopodum* (Maxim.) Fedde (Papaveraceae)	Whole plant	China	Cytotoxicity against H1299, MCF-7, and SMCC-7721 with IC_50_ value of 53.58 ± 5.47 µM, 72.30 ± 1.72 µM, and 73.22 ± 2.35 µM, respectively	[106]
Demethylsonodione **79**	*Hernandia nymphaefolia* (Presi) Kubitzk (Hernandiaceae)	Trunk bark	Taiwan	Exhibited cytotoxic activity against P-388, KB16, A549 (human lung adenocarcinoma), and HT-29 (human colon carcinoma cell lines with ED_50_ value of 0.766, 0.507, 0.223, and 0.772 µg/mL	[110]
Dielsiquinone **3**	*Goniothalamus tamirensis* Pierre ex Finet & Gagnep. (Annonaceae)	Stem bark	Thailand	Showed cytotoxic activity against A549, HT029, MCF7, RPMI and U251 with ED_50_ value of 0.11, 1.12, 0.11, 0.11 and 0.37 µM, respectively	[111]
Glaucine **68**	*Cassytha filiformis* L. (Lauraceae)	Whole plant	Benin	Active compound against HeLa cell line with IC_50_ value of 8.2 µM	[112]
	*Codiaeum variegatum* (L.) Rumph. ex A.Juss. (Euphorbiaceae)	Leaves	Egypt	Showed cytotoxic activity against HepG2, MCF7, HCT116, and A549 cell lines with % of inhibition of cell viability of 38.4%, 46.3%, 66.8%, and 17.3%, respectively (at concentration of 100 µg/mL)	[113]
	*Corydalis turtschaninovii* Bess. (Papaveraceae)	Tuber	Korea	Showed cytotoxic activity against A549, SK-OV-3, SK-MEL-2 and HCT-15 cell lines with IC_50_ value of 26.76 ± 3.82, 21.57 ± 1.01, 20.39 ± 1.45 and 18.63 ± 4.15 µM, respectively	[114]
Isocoreximine **39**	*Guatteria blepharophylla* Mart (Annonaceae)	Bark	Brazil	Showed anti-proliferative activity against UACC-62, MCF-7, NCI-H460, OVCAR-03, PC-3, HT-29, alagnd 786-0 with TGI value of >764.52 µM, and NCI-ADR/RES (TGI 131.50 µM). This compound showed selective activity for ovarian expressing phenotype for multiple drug resistance (NCI-ADR/RES) with a TGI value of 131.50 µM, but was less active than doxorubicin (TGI value of 14.80 µM)	[98]
(+)-Isocorydine **74**	*Cassytha filiformis* L. (Lauraceae)	Whole plant	Benin	Inactive against HeLa cell with IC_50_ > 80 µM	[112]
	*Papaver rhoeas* L. *Papaver rhopalothece* Stapf, *Papaver macrostomum* Boiss. & A.Huet (Papaveraceae)	Aerial parts	Turkey	Nontoxic against normal Vero cell with IC_50_ value of >300 μg/mL	[115]
Lanuginosine **10**	*Magnolia grandiflora* L. (Magnoliaceae)	Leaves	Egypt	Exhibited cytotoxicity against U251 and HEPG2 with IC_50_ value of 4 μg/mL and 2.5 μg/mL, respectively. Lanuginosine 10 was found to be inactive against the HeLa cancer cell.	[116]
Liriodenine **11**	*Anomianthus dulcis* (Dunal) J. Sinclair (Annonaceae)	Stem bark	Thailand	Exhibit the growth of NCIH187, BC, and KB cell lines with IC_50_ values at 1.02, 13.45 and 14.57 µg/mL, respectively	[117]
	*Broussonetia papyrifera* (L.) L′Hér. ex Vent. (Moraceae)	Fruits	China	Exhibit strong cytotoxic effect against A375, BEL-7402, and HeLa cell lines with IC_50_ value of 5.38 ± 0.27, 6.61 ± 0.57, and 5.97 ± 0.39 µg/mL, respectively	[118]
	*Cananga odorata* (Lam.) Hook.f. & Thomson (Annonaceae)	Stem bark	Bangladesh	Show cytotoxic activity based on brine shrimp method with LC_50_ value of 4.89 μg/mL	[119]
	*Cyathostemma argenteum* Wild & R.B.Drumm (Vitaceae)	Roots	Malaysia	Found to be similarly and moderately cytotoxic against MCF-7 ADR MDA-MB435 and MT-1 cells lines with IC_50_ values of 15.6, 16.7, 6.4 and 18.2 µM, respectively	[120]
	*Disepalum plagioneurum* (Diels) D.M.Johnson (Syn *Polyalthia plagioneura* Diels, Annonaceae)	Stem	China	Cytotoxic activity against GSC-7901, K562, and SPCA-1 cell lines with IC_50_ value of of 3.87, 37.61, and 6.19 µM, respectively	[121]
	*Disepalum pulchrum* (King) J.Sinclair (syn *Enicosanthellum pulchrum* (King) Heusden, Annonaceae)	Root	Malaysia	Inhibited CAOV-3 cell growth with IC_50_ value of.3 ± 1.06 µM after 24 h of exposure. Exhibited less activity against SKOV-3 cells, with IC_50_ values of 68.0 ± 1.56 µM.	[122]
	*Goniothalamus gitingensis* Elmer (Annonaceae)	Leaves	Philippines	Effective antiproliferative effects against HUVEC and K-562 cell lines with GI_50_ value of 8.2 ± 0.3 and 6.1 ± 0.8, respectively.	[123]
Liriodenine **11**	*Guatteria aberrans* Erkens & Maas (Syn *Guatteria f riesiana* (W.A. Rodrigues) Erkens & Maas, Annonaceae)	Stem bark	Brazil	Anticancer potent against B16-F10 (mouse melanoma), HepG2 (human hepatocellular carcinoma), HL-60 (human promyelocytic leukemia), and K562 (human chronic myelocytic leukemia) tumor cell lines with IC_50_ values of >10, 8.3, 5.5, and 5.0 μM for the respectively	[124]
	*Guatteria blepharophylla* Mart. (Annonaceae)	Bark	Brazil	Showed anti-proliferative activity against UACC-62, MCF-7, NCI-H460, OVCAR-03, PC-3, HT-29, 786-0 and NCI-ADR/RES with TGI value of 63.02, 37.67, 87.41, 372.18, >909.09, >909.09, >909.09 and >909.09 µM, respectively. This compound undermined positive control doxorubicin against MCF-7 with TGI value of 46.04 µM.	[98]
	*Magnolia duperreana* Pierre (Syn *Kmeria duperreana* (Pierre) Dandy, Magnoliaceae)	Stem bark	Thailand	Found to be active against KB and P388 cell lines with ED_50_ value of of 1.7 and 0.8 µg/mL, respectively	[125]
	*Magnolia floribunda* (Finet & Gagnep.) Figlar (Syn *Michelia floribunda* Finet & Gagnep., Magnoliaceae)	Stem bark	Thailand	Indicated cytotoxic activity against KB and P388 cell lines with with ED_50_ value of <2.5 µg/mL	[126]
	*Michelia compressa* var. formosana (Magnoliaceae)	Heartwood	Taiwan	Exhibited powerful inhibitory activity against TW01, H226, Jurkat, A498, A549, and HT1080 carcinoma cell lines with IC_50_ value of were 8.99, 14.71, 15.7, 4.52, 8.82 and 9.75 μM, respectively	[127]
	*Michelia compressa* var. lanyuensis (Magnoliaceae)	Roots	Taiwan	Possessed cytotoxicity against B16F10 cells after 24 h treatment at high concentration (100 μM) with 80% of cell viability.	[128]
	*Microcos paniculata* L. (Malvaceae)	Branche	Vitenam	Showed low activity against HT-29 cancer cell line with IC_50_ values greater than 10 μM.	[129]
	*Miliusa sinensis* Finet & Gagnep. (Annonaceae)	Leaves and branches	Vietnam	Indicated a good activity against MCF-7, KB, LU and Hep-G2 cancer cell lines with IC_50_ value of 2.89, 2.30, 6.66 and 5.23 μg/mL, respectively	[130]
	*Nelumbo nucifera* Gaertn (Nelumbonaceae)	Leaves	Taiwan	Showed anti-proliferative effects against AGS and DU-145 cell lines with IC_50_ value of >500 and 95.4 ± 0.4 µM, respectively	[99]
	*Polyalthia longifolia* var. pendula (Annonaceae)	Bark	Taiwan	Showed activity against MCF-7 (breast cancer) and MDA-MB-231 cell line with IC_50_ value of 4.46 and 10.28 µg/mL, respectively	[131]
Liriodenine **11**	*Pseuduvaria setosa* (King) J. Sinclair (Annonaceae)	Aerial part	Thailand	Strongly cytotoxic to KB and BC cell lines with IC_50_ 2.4 µg/mL and 2.3 µg/mL, respectively	[132]
	*Saprosma hainanense* Merr. (Rubiaceae)	Stems	China	Exhibit cytotoxic activities against BEL-7402, SGC-7901, and K-562 cell lines with IC_50_ value of 71.7, 33.7, and 197.7 µM, respectively	[105]
	*Stephania dinklagei* (Engl.) Diels (Menispermaceae)	Aerial parts	Ghana	Exhibit cytotoxic activity against KB cell line with IC_50_ value of 26.9 ± 2.4 μM	[107]
	*Stephania dinklagei* (Engl.) Diels (Menispermaceae)	Stem	Ghana	Showed DNA-damaging activity in the yeast bioassay against YCp50 gal, pRAD52 GAL, Prad52 GLU with IC_50_ value of 0.6, 1.5, and 0.5 μg/mL, respectively.	[108]
	*Unonopsis guatterioides* (A.DC.) R.E.Fr.(Sin *Unonopsis buchtienii* R. E.Fries, Annonaceae)	Stem	Bolivia	Possessed cytotoxic bioactivity against Vero cell line with IC_50_ value of 1 μg/mL	[133]
	*Zanthoxylum nitidum* (Roxb.) DC. (Rutaceae)	Stem bark	China	Exhibit cytotoxicity against three human cancer cell lines HT29, A549 and MDA-MB-231 with IC50 values of 9.12, 6.05, and 11.35 μM, respectively	[134]
	*Zanthoxylum nitidum* (Roxb.) DC. (Rutaceae)	Stem bark	Taiwan	Exhibit moderate cytotoxicity against MCF-7, NCI-H460, and SF-268 cancer cell lines with IC_50_ values of 3.19, 2.38, and 2.19, respectively. Liriodenine (11) was the most cytotoxic isolate in *Zanthoxylum nitidum*	[135]
(+)-Nornuciferine **73**	*Guatteria blepharophylla* Mart (Annonaceae)	Stem bark	Brazil	Anti-proliferative activity against MCF-7 NCI-H460 PC-3 HT-29786-0 K562 and NCI-ADR/RES with TGI value of 215.58, 201.99, 542.38, 191.38, 615.23, 153.88 and 255.37 µM, respectively.	[98]
	*Nelumbo nucifera* Gaertn (Nelumbonaceae)	Leaves	Taiwan	Anti-proliferative effects against AGS and DU-145 cell lines with IC_50_ value of >500 µM	[99]
	*Phoebe grandis* (Nees) Merr. (Lauraceae)	Leaves	Malaysia	Cytotoxic activity against NIH/3T3, HeLa and HL-60 with CD_50_ value of 17, 15 and 37 µg/mL, respectively.	[136]
(+)-Reticuline **19**	*Argemone Mexicana* L. (Papaveraceae)	Whole plant	Taiwan	Cytotoxic effects against HONE-1 (96% of control) and NUGC (90% of control) at concentration of 150 µM	[137]
	*Dehaasia longipedicellata* (Ridl.) Kosterm. (Lauraceae)	Stem bark	Malaysia	Cytotoxicity activities against A549 (IC_50_ > 200 µg/mL), A375 (IC_50_ 97.600 µg/mL), and BxPC-3 (IC_50_ 82.570 µg/mL)	[138]
	*Hernandia nymphaefolia* (Presi) Kubitzk (Hernandiaceae)	Trunk bark	Taiwan	Anticancer activity against P-388, KB16, A549 (human lung adenocarcinoma), and HT-29 (human colon carcinoma cell lines with ED_50_ > 50 µg/mL	[139]
Roemerine **49**	*Nelumbo nucifera* Gaertn (Nelumbonaceae)	Leaves	Taiwan	Showed anti-proliferative effects against AGS and DU-145with IC_50_ value of >500 and 95.4 ± 0.4 µM, respectively	[99]
(−)-Stepholidine **20**	*Polyalthia longifolia* (Sonn.) Thwaites (Annonaceae)	Bark	Taiwan	Activity against MCF-7 (breast cancer) cell line with IC_50_ value of 16.56 µg/mL	[131]
Squamolone **82**	*Artabotrys hexapetalus* (L.f.) Bhandari (Syn *Artabotrys uncinatus* (Lam) Merr., Annonaceae)	Roots, stems, and leaves	Taiwan	Showed significant activity against Hep G2 and 2,2,15 cell lines with IC_50_ value of 2.8 and 1.6 µg/mL, respectively	[102]

## References

[1] The Plant List Version 1. http://www.theplantlist.org/.

[2] Badrie N., Schauss A.G., Watson R.R., Preedy V.R. (2010). Soursop (*Annona muricata* L.): Composition, nutritional value, medicinal uses, and toxicology. Bioactive Foods in Promoting Health.

[3] Mishra S., Ahmad S., Kumar N., Sharma B.K. (2013). *Annona muricata* (the cancer killer): A Review. Glob. J. Pharm. Res..

[4] Oliveira B.H., Sant’Ana A.E.G., Bastos D.Z.L. (2002). Determination of the diterpenoid, kaurenoic acid, in *Annona glabra* by HPLC. Phytochem. Anal..

[5] Barbalho S., de Goulart R., Vasques Farinazzi-Machado F., da Soares de Souza M., Santos Bueno P., Guiguer E., Araujo A., Groppo M. (2012). *Annona* sp: Plants with Multiple Applications as Alternative Medicine - A Review. Curr. Bioact. Compd..

[6] Asare G.A., Afriyie D., Ngala R.A., Abutiate H., Doku D., Mahmood S.A., Rahman H. (2015). Antiproliferative activity of aqueous leaf extract of *Annona muricata* L. on the prostate, BPH-1 cells, and some target genes. Integr. Cancer Ther..

[7] Quílez A.M., Fernández-Arche M.A., García-Giménez M.D., De la Puerta R. (2018). Potential therapeutic applications of the genus Annona: Local and traditional uses and pharmacology. J. Ethnopharmacol..

[8] Coria-Téllez A.V., Montalvo-Gónzalez E., Yahia E.M., Obledo-Vázquez E.N. (2018). *Annona muricata*: A comprehensive review on its traditional medicinal uses, phytochemicals, pharmacological activities, mechanisms of action and toxicity. Arab. J. Chem..

[9] Morton J.F., Dowling C.F. (1987). Fruits of Warm Climates.

[10] Jansen P.C.M., Jukema J., Oyen L.P.A., van Lingen T.G., Verheij E.W.M., Coronel R.E. (1991). *Annona reticulata* L.. Plant Resources of South-East Asia No. 2: Edible fruits and nuts.

[11] Karou S.D., Tchacondo T., Djikpo Tchibozo M.A., Abdoul-Rahaman S., Anani K., Koudouvo K., Batawila K., Agbonon A., Simpore J., de Souza C. (2011). Ethnobotanical study of medicinal plants used in the management of diabetes mellitus and hypertension in the Central Region of Togo. Pharm. Biol..

[12] Dos S.A.F., Sant’Ana A.E. (2000). The molluscicidal activity of plants used in Brazilian folk medicine. Phytomedicine.

[13] Syamsuhidayat S., Hutapea J.R. (1991). Inventaris Tanaman Obat Indonesia.

[14] DeFilipps R.A., Maina S.L., Crepin J. (2004). Medicinal Plants of the Guianas (Guyana, Surinam, French Guiana).

[15] González-Trujano M.E., Navarrete A., Reyes B., Hong E. (1998). Some pharmacological effects of the ethanol extract of leaves of *Annona diversifolia* on the central nervous system in mice. Phyther. Res..

[16] Oliveira da Cruz P.E., Costa E.V., de S. Moraes V.R., de L. Nogueira P.C., Vendramin M.E., Barison A., Ferreira A.G., do N. Prata A.P. (2011). Chemical constituents from the bark of *Annona salzmannii* (Annonaceae). Biochem. Syst. Ecol..

[17] Ribeiro da Silva L.M., Teixeira de Figueiredo E.A., Ricardo N.M.P.S., Vieira I.G.P., Wilane de Figueiredo R., Brasil I.M., Gomes C.L. (2014). Quantification of bioactive compounds in pulps and by-products of tropical fruits from Brazil. Food Chem..

[18] Nugraha A.S., Haritakun R., Lambert J.M., Dillon C.T., Keller P.A. (2019). Alkaloids from the root of Indonesian *Annona muricata* L.. Nat. Prod. Res..

[19] Rupprecht J.K., Hui Y.-H., McLaughlin J.L. (1990). Annonaceous Acetogenins: A Review. J. Nat. Prod..

[20] Fang X.-P., Rieser M.J., Gu Z.-M., Zhao G.-X., McLaughlin J.L. (1993). Annonaceous acetogenins: An updated review. Phytochem. Anal..

[21] Feras Q.A., Liu A., McLaughlin J.L. (1999). Annonaceous Acetogenins: Recent Progress. J. Nat. Prod..

[22] Zeng L., Ye Q., Oberlies H., Shi G., Gu Z.-M., He K., McLaughlin J.L. (1996). Recent advances in Annonaceous acetogenins. Nat. Prod. Rep..

[23] Moghadamtousi S.Z., Fadaeinasab M., Nikzad S., Mohan G., Ali H.M., Abdul Kadir H. (2015). *Annona muricata* (Annonaceae): A review of its traditional uses, isolated acetogenins and biological activities. Int. J. Mol. Sci..

[24] Reyes F.R., Santos A.C. (1931). Isolation of anonaine from *Anona squamosa* Linn. Philipp. J. Sci..

[25] De Oliveira A.B., De Oliveira G.G., Carazza F., Maia J.G.S. (1987). Geovanine, a new azaanthracene alkaloid from *Annona ambotay* Aubl. Phytochemistry.

[26] Rabelo S.V., Costa E.V., Barison A., Dutra L.M., Nunes X.P., Tomaz J.C., Oliveira G.G., Lopes N.P., de F.C. Santos M., da Silva Almeida J.R.G. (2015). Alkaloids isolated from the leaves of atemoya (*Annona cherimola* × *Annona squamosa*). Rev. Bras. Farmacogn..

[27] Raju D.U., Babu K.S., Ravada S.C.R., Golakoti T. (2015). Isoquinoline alkaloid, flavonoids and a triol from leaves of *Annona cherimola*. J. Appl. Chem. (Lumami, India).

[28] Villar A., Mares M., Rios J.L., Cortes D. (1985). Alkaloids from *Annona cherimolia* leaves. J. Nat. Prod..

[29] Rios J.L., Cortes D., Valverde S. (1989). Acetogenins, aporphinoids, and azaanthraquinone from *Annona cherimolia* seeds. Planta Med..

[30] Villar del Fresno A., Rios Canavate J.L. (1983). Alkaloids from *Annona cherimolia* seed. J. Nat. Prod..

[31] Chen C.-Y., Chang F.-R., Wu Y.-C. (1997). Cherimoline, a novel alkaloid from the stems of *Annona cherimola*. Tetrahedron Lett..

[32] Chen C.Y., Chang F.R., Pan W.B., Wu Y.C. (2001). Four alkaloids from *Annona cherimola*. Phytochemistry.

[33] Simeon S., Rios J.L., Villar A. (1989). Alkaloids from *Annona cherimolia* (Mill.) stem bark. Plant. Med. Phytother..

[34] Martinez-Vazquez M., De la Cueva Lozano D.G., Estrada-Reyes R., Gonzalez-Lugo N.M., Ramirez Apan T., Heinze G. (2005). Bio-guided isolation of the cytotoxic corytenchine and isocoreximine from roots of *Annona cherimolia*. Fitoterapia.

[35] de la Cruz Chacon I., Gonzalez-Esquinca A.R. (2012). Liriodenine alkaloid in *Annona diversifolia* during early development. Nat. Prod. Res..

[36] Chang F.-R., Chen C.-Y., Hsieh T.-J., Cho C.-P., Wu Y.-C. (2000). Chemical constituents from *Annona glabra* III. J. Chin. Chem. Soc..

[37] Riley-Saldana C.A., del R. Cruz-Ortega M., Martinez Vazquez M., De-la-Cruz-Chacon I., Castro-Moreno M., Gonzalez-Esquinca A.R. (2017). Acetogenins and alkaloids during the initial development of *Annona muricata* L. (Annonaceae). Zeitschrift fuer Naturforschung C.

[38] Yang T.-H., Chen C.-M., Kuan S.-S. (1971). Alkaloids of *Annona glabra*. I. Isolation of (−)-*N*-methylactinodaphnine. J. Chin. Chem. Soc..

[39] Yang T.-H., Chen C.-M. (1973). Studies on the alkaloids of *Anona glabra*. II. T’ai-wan Yao Hsueh Tsa Chih.

[40] Yang T.-H., Chen C.-M. (1974). Studies on the alkaloids of *Anona glabra* L. II. Proc. Natl. Sci. Counc., Part. 2.

[41] Wu Y.C., Chang G.Y., Duh C.Y., Wang S.K. (1993). Cytotoxic alkaloids of *Annona montana*. Phytochemistry.

[42] Leboeuf M., Cave A., Forgacs P., Tiberghien R., Provost J., Touche A., Jacquemin H. (1982). Alkaloids of the genus Annona. XL. Chemical and pharmacological study of alkaloids from *Annona montana* Macf. Plant. Med. Phytother..

[43] Yokomori Y., Sekido K., Wu T.S., Tien H.J., Hirokawa S. (1982). The crystal and molecular structure of 1-(2-amino-4-pyrimidinyl)-β-carboline. Bull. Chem. Soc. Jpn..

[44] Magadula J.J., Innocent E., Otieno J.N. (2009). Mosquito larvicidal and cytotoxic activities of 3 Annona species and isolation of active principles. J. Med. Plants Res..

[45] Matsushige A., Kotake Y., Matsunami K., Otsuka H., Ohta S., Takeda Y. (2012). Annonamine, a new aporphine alkaloid from the leaves of *Annona muricata*. Chem. Pharm. Bull..

[46] Fofana S., Keita A., Balde S., Ziyaev R., Aripova S.F. (2012). Alkaloids from leaves of *Annona muricata*. Chem. Nat. Compd..

[47] Fofana S., Ziyaev R., Abdusamatov A., Zakirov S.K. (2011). Alkaloids from *Annona muricata* leaves. Chem. Nat. Compd..

[48] Leboeuf M., Legueut C., Cave A., Desconclois J.F., Forgacs P., Jacquemin H. (1981). [Alkaloids of Annonaceae. XXIX. Alkaloids of *Annona muricata*]. Planta Med..

[49] Laprevote O., Leboeuf M., Cave A., Provost J., Forgacs P., Jacquemin H. (1988). Alkaloids of the Annonaceae. 88. Alkaloids of *Annona paludosa* Aublet. Plant. Med. Phytother..

[50] Chang F.-R., Chen K.-S., Ko F.-N., Teng C.-M., Wu Y.-C. (1995). Bioactive alkaloids from *Annona reticulata*. Chin. Pharm. J..

[51] Yang T.H., Cheng M.Y. (1987). The alkaloids of *Annona reticulata* L. II. T’ai-wan Yao Hsueh Tsa Chih.

[52] Xu L., Li K., Sun N., Kong J. (1992). Alkaloids of *Annona reticulata*. Zhongguo Zhongyao Zazhi.

[53] Campos F.R., Batista R.L., Batista C.L., Costa E.V., Barison A., dos Santos A.G., Pinheiro M.L.B. (2008). Isoquinoline alkaloids from leaves of *Annona sericea* (Annonaceae). Biochem. Syst. Ecol..

[54] Pinto N.C.C., Silva J.B., Menegati L.M., Guedes M.C.M.R., Scio E., Fabri R.L., Marques L.B., Souza-Fagundes E.M.D.E., Silva T.P.D.A., Melo R.C.N.D.E. (2017). Cytotoxicity and bacterial membrane destabilization induced by *Annona squamosa* L. extracts. An. Acad. Bras. Cienc..

[55] Philipov S., Kande K.M., Machev K. (1995). Alkaloids of *Annona senegalensis*. Fitoterapia.

[56] Fofana S., Ziyaev R., Diallo S.K., Camara M., Aripova S.F. (2013). Alkaloids of *Annona senegalensis*. Chem. Nat. Compd..

[57] Bhakuni D.S., Tewari S., Dhar M.M. (1972). Aporphine alkaloids of *Annona squamosa*. Phytochemistry.

[58] Bhaumik P.K., Mukherjee B., Juneau J.P., Bhacca N.S., Mukerjee R. (1979). Alkaloids from leaves of *Annona squamosa*. Phytochemistry.

[59] You M., Mahinda Wickramaratne D.B., Silva G.L., Chai H., Chagwedera T.E., Farnsworth N.R., Cordell G.A., Kinghorn A.D., Pezzuto J.M. (1995). (-)-Roemerine, an aporphine alkaloid from *Annona senegalensis* that reverses the multidrug-resistance phenotype with cultured cells. J. Nat. Prod..

[60] Yang Y.-L., Chang F.-R., Wu Y.-C. (2004). Annosqualine: A novel alkaloid from the stems of *Annona squamosa*. Helv. Chim. Acta.

[61] Yang T.-H., Chen C.-M. (1970). Constituents of *Annona squamosa*. J. Chin. Chem. Soc..

[62] Pimenta L.P.S., Garcia G.M., do V. Goncalves S.G., Dionisio B.L., Braga E.M., Mosqueira V.C.F. (2014). In vivo antimalarial efficacy of acetogenins, alkaloids and flavonoids enriched fractions from *Annona crassiflora* Mart. Nat. Prod. Res..

[63] Kamaraj C., Kaushik N.K., Mohanakrishnan D., Elango G., Bagavan A., Zahir A.A., Rahuman A.A., Sahal D. (2012). Antiplasmodial potential of medicinal plant extracts from Malaiyur and Javadhu hills of South India. Parasitol Res..

[64] Somsak V., Polwiang N., Chachiyo S. (2016). In vivo antimalarial activity of *Annona muricata* leaf extract in mice infected with *Plasmodium berghei*. J. Pathog..

[65] Meira C.S., Guimaraes E.T., Macedo T.S., da Silva T.B., Menezes L.R.A., Costa E.V., Soares M.B.P. (2015). Chemical composition of essential oils from *Annona vepretorum* Mart. and *Annona squamosa* L. (Annonaceae) leaves and their antimalarial and trypanocidal activities. J. Essent. Oil Res..

[66] Kamaraj C., Bagavan A., Elango G., Zahir A.A., Rajakumar G., Marimuthu S., Santhoshkumar T., Abdul Rahuman A. (2011). Larvicidal activity of medicinal plant extracts against *Anopheles subpictus* & *Culex tritaeniorhynchus*. Indian J. Med. Res..

[67] Kihampa C., Joseph C.C., Nkunya M.H.H., Magesa S.M., Hassanali A., Heydenreich M., Kleinpeter E. (2009). Larvicidal and IGR activity of extract of Tanzanian plants against malaria vector mosquitoes. J. Vector Borne Dis..

[68] Osorio E., Arango G.J., Jimenez N., Alzate F., Ruiz G., Gutierrez D., Paco M.A., Gimenez A., Robledo S. (2007). Antiprotozoal and cytotoxic activities in vitro of Colombian Annonaceae. J. Ethnopharmacol.

[69] Yamthe L.R.T., Fokou P.V.T., Mbouna C.D.J., Keumoe R., Ndjakou B.L., Djouonzo P.T., Mfopa A.N., Legac J., Tsabang N., Gut J. (2015). Extracts from *Annona muricata* L. and *Annona reticulata* L. (Annonaceae) potently and selectively inhibit Plasmodium falciparum. Medicines.

[70] Garcia Diaz J., Tuenter E., Escalona Arranz J.C., Llaurado Maury G., Cos P., Pieters L. (2019). Antimicrobial activity of leaf extracts and isolated constituents of *Croton linearis*. J. Ethnopharmacol..

[71] Mollataghi A., Coudiere E., Hadi A.H.A., Mukhtar M.R., Awang K., Litaudon M., Ata A. (2012). Anti-acetylcholinesterase, anti-α-glucosidase, anti-leishmanial and anti-fungal activities of chemical constituents of Beilschmiedia species. Fitoterapia.

[72] de Lima J.P.S., Pinheiro M.L.B., Santos A.M.G., Pereira J.L.S., Santos D.M.F., Barison A., Silva-Jardim I., Costa E. (2012). V In vitro antileishmanial and cytotoxic activities of *Annona mucosa* (Annonaceae). Rev. Virtual Quim..

[73] de Omena M.C., Navarro D.M.A.F., de Paula J.E., Luna J.S., Ferreira de Lima M.R., Sant’Ana A.E.G. (2007). Larvicidal activities against *Aedes aegypti* of some Brazilian medicinal plants. Bioresour. Technol..

[74] Rodrigues A.M., De Paula J.E., Degallier N., Molez J.E., Espindola L.S. (2006). Larvicidal activity of some Cerrado plant extracts against *Aedes aegypti*. J. Am. Mosq Control. Assoc..

[75] Hoe P.K., Yiu P.H., Eea G.C.L., Wong S.C., Rajan A., Bong C.F.J. (2010). Biological Activity of *Annona muricata* Seed Extracts. Malaysian J. Sci..

[76] Das N.G., Goswami D., Rabha B. (2007). Preliminary evaluation of mosquito larvicidal efficacy of plant extracts. J. Vector Borne Dis.

[77] Panda S.K., Mohanta Y.K., Padhi L., Park Y.-H., Mohanta T.K., Bae H. (2016). Large scale screening of ethnomedicinal plants for identification of potential antibacterial compounds. Molecules.

[78] Fu L., Lu W., Zhou X. (2016). Phenolic compounds and in vitro antibacterial and antioxidant activities of three tropic fruits: Persimmon, guava, and sweetsop. Biomed. Res. Int..

[79] Wu T.-S., Jong T.-T., Tien H.-J., Kuoh C.-S., Furukawa H., Lee K.-H. (1987). Annoquinone-A, an antimicrobial and cytotoxic principle from *Annona montana*. Phytochemistry.

[80] Takahashi J.A., Pereira C.R., Pimenta L.P.S., Boaventura M.A.D., Silva L.G.F.E. (2006). Antibacterial activity of eight Brazilian annonaceae plants. Nat. Prod. Res..

[81] Bories C., Loiseau P., Cortes D., Myint S.H., Hocquemiller R., Gayral P., Cave A., Laurens A. (1991). Antiparasitic activity of Annona muricata and *Annona cherimolia* seeds. Planta Med..

[82] Bento E.B., Matias E.F.F., Brito F.E., Oliveira D.R., Coutinho H.D.M., Costa J.G.M., Kerntopf M.R., Menezes I.R.A. (2012). Association between food and drugs: Antimicrobial and synergistic activity of *Annona muricata* L.. Int. J. Food Prop..

[83] Tsobou R., Mapongmetsem P.-M., Voukeng K.I., Van Damme P. (2015). Phytochemical screening and antibacterial activity of medicinal plants used to treat typhoid fever in Bamboutos division, West Cameroon. J. Appl. Pharm. Sci..

[84] Dzotam J.K., Touani F.K., Kuete V. (2016). Antibacterial activities of the methanol extracts of *Canarium schweinfurthii* and four other Cameroonian dietary plants against multi-drug resistant Gram-negative bacteria. Saudi J. Biol. Sci..

[85] Essama S.H.R., Nyegue M.A., Foe C.N., Silihe K.K., Tamo S.P.B., Etoa F.X. (2015). Antibacterial and antioxidant activities of hydro-ehanol extracts of barks, leaves and stems of *Annona muricata*. Am. J. Pharmacol. Sci..

[86] Darji B., Ratani J., Doshi M., Kothari V. (2012). In vitro antimicrobial activity in certain plant products/seed extracts against selected phytopathogens. Res. Pharm..

[87] Mohamad N., Majid E.-M., Falah A., Layla C., Akram H., Ali C., Hassan R. (2017). Antibacterial, antioxidant and antiproliferative activities of the hydroalcoholic extract of the Lebanese *Annona squamosa* L. seeds. Int. Res. J. Pharm..

[88] More G., Tshikalange T.E., Lall N., Botha F., Meyer J.J.M. (2008). Antimicrobial activity of medicinal plants against oral microorganisms. J. Ethnopharmacol.

[89] Costa E.V., da Cruz P.E.O., de Lourenço C.C., de Souza Moraes V.R., de Lima Nogueira P.C., Salvador M.J. (2012). Antioxidant and antimicrobial activities of aporphinoids and other alkaloids from the bark of *Annona salzmannii* A. DC. (Annonaceae). Nat. Prod. Res..

[90] Bettarini F., Borgonovi G.E., Fiorani T., Gagliardi I., Caprioli V., Massardo P., Ogoche J.I.J., Hassanali A., Nyandat E., Chapya A. (1993). Antiparasitic compounds from East African plants: Isolation and biological activity of anonaine, matricarianol, canthin-6-one and caryophyllene oxide. Insect Sci. Its Appl..

[91] Rao G.-X., Zhang S., Wang H.-M., Li Z.-M., Gao S., Xu G.-L. (2009). Antifungal alkaloids from the fresh rattan stem of *Fibraurea recisa Pierre*. J. Ethnopharmacol..

[92] Ma C., Du F., Yan L., He G., He J., Wang C., Rao G., Jiang Y., Xu G. (2015). Potent activities of roemerine against *Candida albicans* and the underlying mechanisms. Molecules.

[93] Morteza-Semnani K., Amin G., Shidfar M.R., Hadizadeh H., Shafiee A. (2003). Antifungal activity of the methanolic extract and alkaloids of *Glaucium oxylobum*. Fitoterapia.

[94] Wu C.-C., Wu C.-L., Huang S.-L., Chang H.-T. (2012). Antifungal activity of Liriodenine from *Michelia formosana* heartwood against wood-rotting fungi. Wood Sci. Technol..

[95] Ye Y., Liu J., Liu X., Qiu J., Min H., Zheng R., Xu H., Li H., Zhan R., Chen W. (2013). Antibacterial constituents from roots of *Zanthoxylum nitidum*. Zhongcaoyao.

[96] Li C., Lee D., Graf T.N., Phifer S.S., Nakanishi Y., Riswan S., Setyowati F.M., Saribi A.M., Soejarto D.D., Farnsworth N.R. (2009). Bioactive constituents of the stem bark of *Mitrephora glabra*. J. Nat. Prod..

[97] Ibrahim S.R.M., Mohamed G.A., Zayed M.F., Sayed H.M. (2015). Ingenines A and B, Two new alkaloids from the Indonesian sponge *Acanthostrongylophora ingens*. Drug Res..

[98] Costa E.V., Marques F. (2011). de A.; Pinheiro, M.L.B.; Braga, R.M.; Delarmelina, C.; Duarte, M.C.T.; Ruiz, A.L.T.G.; Ernesto de Carvalho, J.; Maia, B.H.L.N.S. Chemical constituents isolated from the bark of *Guatteria blepharophylla* (Annonaceae) and their antiproliferative and antimicrobial activities. J. Braz. Chem. Soc..

[99] Liu C.-M., Kao C.-L., Wu H.-M., Li W.-J., Huang C.-T., Li H.-T., Chen C.-Y. (2014). Antioxidant and anticancer aporphine alkaloids from the leaves of *Nelumbo nucifera* Gaertn. cv. Rosa-plena. Molecules.

[100] Chen C.-Y., Liu T.-Z., Tseng W.-C., Lu F.-J., Hung R.-P., Chen C.-H., Chen C.-H. (2008). (-)-Anonaine induces apoptosis through Bax- and caspase-dependent pathways in human cervical cancer (HeLa) cells. Food Chem. Toxicol..

[101] Ibrahim S.R.M., Ebel R., Ebel R., Proksch P. (2008). Acanthomine A, a new pyrimidine-β-carboline alkaloid from the sponge *Acanthostrongylophora ingens*. Nat. Prod. Commun..

[102] Hsieh T.-J., Chang F.-R., Chia Y.-C., Chen C.-Y., Lin H.-C., Chiu H.-F., Wu Y.-C. (2001). The alkaloids of *Artabotrys uncinatus*. J. Nat. Prod..

[103] Hsieh T.-J., Chang F.-R., Chia Y.-C., Chen C.-Y., Chiu H.-F., Wu Y.-C. (2001). Cytotoxic Constituents of the Fruits of *Cananga odorata*. J. Nat. Prod..

[104] Nordin N., Majid N.A., Mohan S., Dehghan F., Karimian H., Rahman M.A., Ali H.M., Hashim N.M. (2016). Cleistopholine isolated from *Enicosanthellum pulchrum* exhibits apoptogenic properties in human ovarian cancer cells. Phytomedicine.

[105] Wang L., Chen G.-Y., Han C.-R., Yuan Y., Yang B., Zhang Y., Wang J., Zhong X.-Q., Huang X. (2011). Two novel alkaloids from the stem of *Saprosma hainanense* and their cytotoxic activities in vitro. Chem. Pharm. Bull..

[106] Sun R., He Q., Deng Z., Geng Z., Jiang H., Zhang W., Yang K., Du S., Wang C., Fan L. (2014). Cytotoxicity of Aporphine, Protoberberine, and Protopine Alkaloids from *Dicranostigma leptopodum* (Maxim.) Fedde. Evid. Based. Complement. Alternat. Med..

[107] Del Rayo Camacho M., Kirby G.C., Warhurst D.C., Croft S.L., Phillipson J.D. (2000). Oxoaporphine alkaloids and quinones from *Stephania dinklagei* and evaluation of their antiprotozoal activities. Planta Med..

[108] Goeren A.C., Zhou B., Kingston D.G.I. (2003). Cytotoxic and DNA damaging activity of some aporphine alkaloids from *Stephania dinklagei*. Planta Med..

[109] Rong L., Hu D., Wang W., Zhao R., Xu X., Jing W. (2016). Alkaloids from root tubers of *Stephania kwangsiensis* H.S.Lo and their effects on proliferation and apoptosis of lung NCI-H446 cells. Biomed. Res..

[110] Chen J.J., Ishikawa T., Duh C.Y., Tsai I.L., Chen I.S. (1996). New dimeric aporphine alkaloids and cytotoxic constituents of *Hernandia nymphaefolia*. Planta Med..

[111] Soonthornchareonnon N., Suwanborirux K., Bavovada R., Patarapanich C., Cassady J.M. (1999). New cytotoxic 1-azaanthraquinones and 3-aminonaphthoquinone from the stem bark of *Goniothalamus marcanii*. J. Nat. Prod..

[112] Hoet S., Stevigny C., Block S., Opperdoes F., Colson P., Baldeyrou B., Lansiaux A., Bailly C., Quetin-Leclercq J. (2004). Alkaloids from *Cassytha filiformis* and related aporphines: Antitrypanosomal activity, cytotoxicity, and interaction with DNA and topoisomerases. Planta Med..

[113] Hassan E.M., Hassan R.A., Salib J.Y., Mohamed S.M., El-Toumy S.A. (2013). Chemical constituents and cytotoxic activity of *Codiaeum variegatum* CV. petra. J. Appl. Sci. Res..

[114] Kim K.H., Piao C.J., Choi S.U., Son M.W., Lee K.R. (2010). New cytotoxic tetrahydroprotoberberine-aporphine dimeric and aporphine alkaloids from *Corydalis turtschaninovii*. Planta Med..

[115] Demirgan R., Karagoz A., Pekmez M., Onay-Ucar E., Artun F.T., Gurer C., Mat A. (2016). In vitro anticancer activity and cytotoxicity of some papaver alkaloids on cancer and normal cell lines. African J. Tradit. Complement. Altern. Med..

[116] Mohamed S.M., Hassan E.M., Ibrahim N.A. (2010). Cytotoxic and antiviral activities of aporphine alkaloids of *Magnolia grandiflora* L.. Nat. Prod. Res..

[117] Ubonopas L., Wongsinkongman P., Chuakul W., Suwanborirux K., Lee K.H., Soonthornchareonnon N. (2014). Bioactive flavonoids and alkaloids from *Anomianthus dulcis* (Dunal) J. Sinclair stem bark. Mahidol Univ. J. Pharm. Sci..

[118] Pang S.-Q., Wang G.-Q., Lin J., Diao Y., Xu R. (2014). Cytotoxic activity of the alkaloids from *Broussonetia papyrifera* fruits. Pharm. Biol..

[119] Rahman M.M., Lopa S.S., Sadik G., Harun-or-Rashid, Islam R., Khondkar P., Alam A.H.M.K., Rashid M.A. (2005). Antibacterial and cytotoxic compounds from the bark of *Cananga odorata*. Fitoterapia.

[120] Khamis S., Bibby M.C., Brown J.E., Cooper P.A., Scowen I., Wright C.W. (2004). Phytochemistry and preliminary biological evaluation of *Cyathostemma argenteum*, a Malaysian plant used traditionally for the treatment of breast cancer. Phyther. Res..

[121] Liu B., Jian L., Chen G., Song X., Han C., Wang J. (2014). Chemical constituents and in vitro anticancer cytotoxic activities of *Polyalthia plagioneura*. Chem. Nat. Compd..

[122] Nordin N., Abdul Majid N., Hashim N.M., Abd Rahman M., Hassan Z., Ali H.M. (2015). Liriodenine, an aporphine alkaloid from *Enicosanthellum pulchrum*, inhibits proliferation of human ovarian cancer cells through induction of apoptosis via the mitochondrial signaling pathway and blocking cell cycle progression. Drug Des. Devel. Ther..

[123] Macabeo A.P.G., Lopez A.D.A., Schmidt S., Heilmann J., Dahse H.-M., Alejandro G.J.D., Franzblau S.G. (2014). Antitubercular and cytotoxic constituents from *Goniothalamus gitingensis*. Rec. Nat. Prod..

[124] Costa E.V., Pinheiro M.L.B., Maia B.H.L.N.S., Marques F.A., Ruiz A.L.T.G., Marchetti G.M., de Carvalho J.E., Soares M.B.P., Costa C.O.S., Galvao A.F.C. (2016). 7,7-Dimethylaporphine and Other Alkaloids from the Bark of *Guatteria friesiana*. J. Nat. Prod..

[125] Dong X., Mondranondra I.O., Che C.T., Fong H.H.S., Farnsworth N.R. (1989). Kmeriol and other aromatic constituents of *Kmeria duperreana*. Pharm. Res..

[126] Mondranondra I.O., Che C.T., Rimando A.M., Vajrodaya S., Fong H.H.S., Farnsworth N.R. (1990). Sesquiterpene lactones and other constituents from a cytotoxic extract of *Michelia floribunda*. Pharm. Res..

[127] Chan Y.-Y., Juang S.-H., Huang G.-J., Liao Y.-R., Chen Y.-F., Wu C.-C., Chang H.-T., Wu T.-S. (2014). The constituents of *Michelia compressa* var. formosana and their bioactivities. Int. J. Mol. Sci..

[128] Chu C.-W., Liu C.-M., Chung M.-I., Chen C.-Y. (2015). Biofunctional constituents from *Michelia compressa* var. lanyuensis with anti-melanogenic properties. Molecules.

[129] Still P.C., Yi B., Gonzalez-Cestari T.F., Pan L., Pavlovicz R.E., Chai H.-B., Ninh T.N., Li C., Soejarto D.D., McKay D.B. (2013). Alkaloids from *Microcos paniculata* with Cytotoxic and Nicotinic Receptor Antagonistic Activities. J. Nat. Prod..

[130] Thuy T.T.T., Quan T.D., Nguyen T.H.A., Sung T.V. (2011). A new hydrochalcone from *Miliusa sinensis*. Nat. Prod. Res..

[131] Chang F.-R., Hwang T.-L., Yang Y.-L., Li C.-E., Wu C.-C., Issa H.H., Hsieh W.-B., Wu Y.-C. (2006). Anti-inflammatory and cytotoxic diterpenes from formosan *Polyalthia longifolia* var. pendula. Planta Med..

[132] Wirasathien L., Boonarkart C., Pengsuparp T., Suttisri R. (2006). Biological activities of alkaloids from *Pseuduvaria setosa*. Pharm. Biol..

[133] Waechter A.-I., Cave A., Hocquemiller R., Bories C., Munoz V., Fournet A. (1999). Antiprotozoal activity of aporphine alkaloids isolated from *Unonopsis buchtienii* (Annonaceae). Phyther. Res..

[134] Zhao L.-N., Wang J., Wang Z., Tan N.-H. (2018). Chemical and cytotoxic constituents of *Zanthoxylum nitidum*. Zhongguo Zhong Yao Za Zhi.

[135] Yang C.-H., Cheng M.-J., Lee S.-J., Yang C.-W., Chang H.-S., Chen I.-S. (2009). Secondary metabolites and cytotoxic activities from the stem bark of *Zanthoxylum nitidum*. Chem. Biodivers..

[136] Amna U., Hasnan M.H.H., Ahmad K., Abdul Manaf A., Awang K., Nafiah M.A. (2015). In vitro cytotoxic of aporphine and proaporphine alkaloids from phoebe grandis (Ness) merr. Int. J. Pharm. Sci. Rev. Res..

[137] Chang Y.-C., Chang F.-R., Khalil A.T., Hsieh P.-W., Wu Y.-C. (2003). Cytotoxic benzophenanthridine and benzylisoquinoline alkaloids from *Argemone mexicana*. Z. Naturforsch. C..

[138] Zahari A., Cheah F.K., Mohamad J., Sulaiman S.N., Litaudon M., Leong K.H., Awang K. (2014). Antiplasmodial and antioxidant isoquinoline alkaloids from *Dehaasia longipedicellata*. Planta Med..

[139] Chen I.S., Chen J.J., Duh C.Y., Tsai J.L., Chang C.T. (1997). New aporphine alkaloids and cytotoxic constituents of *Hernandia nymphaefolia*. Planta Med..

